# Does the crystal structure of vanadium nitrogenase contain a reaction intermediate? Evidence from quantum refinement

**DOI:** 10.1007/s00775-020-01813-z

**Published:** 2020-08-27

**Authors:** Lili Cao, Octav Caldararu, Ulf Ryde

**Affiliations:** Department of Theoretical Chemistry, Chemical Centre, Lund University, P. O. Box 124, 221 00 Lund, Sweden

**Keywords:** Nitrogenase, QM/MM, S2B dissociation, Nitrogen fixation, Quantum refinement

## Abstract

**Abstract:**

Recently, a crystal structure of V-nitrogenase was presented, showing that one of the µ_2_ sulphide ions in the active site (S2B) is replaced by a lighter atom, suggested to be NH or NH_2_, i.e. representing a reaction intermediate. Moreover, a sulphur atom is found 7 Å from the S2B site, suggested to represent a storage site for this ion when it is displaced. We have re-evaluated this structure with quantum refinement, i.e. standard crystallographic refinement in which the empirical restraints (employed to ensure that the final structure makes chemical sense) are replaced by more accurate quantum–mechanical calculations. This allows us to test various interpretations of the structure, employing quantum–mechanical calculations to predict the ideal structure and to use crystallographic measures like the real-space *Z*-score and electron-density difference maps to decide which structure fits the crystallographic raw data best. We show that the structure contains an OH^−^-bound state, rather than an N_2_-derived reaction intermediate. Moreover, the structure shows dual conformations in the active site with ~ 14% undissociated S2B ligand, but the storage site seems to be fully occupied, weakening the suggestion that it represents a storage site for the dissociated ligand.

**Graphic abstract:**

**Electronic supplementary material:**

The online version of this article (10.1007/s00775-020-01813-z) contains supplementary material, which is available to authorized users.

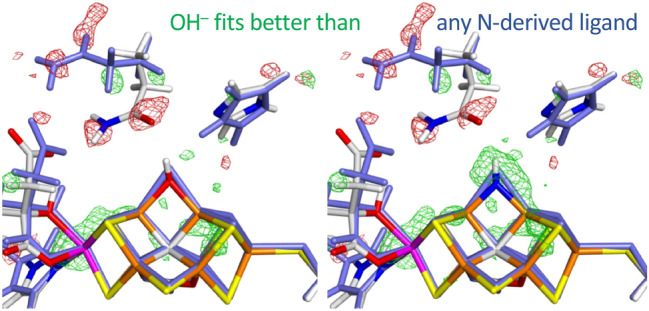

## Introduction

The atmosphere of Earth contains 78% N_2_, but nitrogen is still a limiting element for most plant life. The reason for this is that the triple bond in N_2_ is very strong, making N_2_ highly inert [1, 2]. In 1909, Fritz Haber designed a procedure to form ammonia from N_2_ and H_2_, employing high temperature and pressure. It was adapted for industrial use by Carl Bosch at BASF and is today known as the Haber–Bosch process. It is currently one of the most important industrial processes, consuming 1–2% of the world’s total energy supplies and it is a main reason for the human population explosion during the twentieth century, by providing abundant access to artificial fertilisers [3, 4].

In nature, a single enzyme, nitrogenase (EC 1.18/19.6.1), can convert N_2_ to ammonia at ambient pressure and temperature [1, 5, 6]. It is found in a few bacteria and archaea, but many higher plants, e.g. legumes, rice and alder, live in symbiosis with such organisms, obtaining bio-available nitrogen in exchange for carbohydrates. The nitrogenase reaction is quite demanding, requiring at least 16 molecules of ATP for each nitrogen molecule processed [1, 5, 6]:

1 $${\text{N}}_{{2}} + {\text{8e}}^{-} + {\text{8H}}^{ + } + {\text{16ATP}} \longrightarrow {\text{2NH}}_{{3}} + {\text{H}}_{{2}} + {\text{16ADP}} + {\text{16P}}_{{\text{i}}} .$$

It is notable that H_2_ is a compulsory by-product. The mechanism of the nitrogenases has been extensively studied by biochemical, kinetic, spectroscopic and computational methods [1, 5, 7–9]. However, many details of the mechanism are still unknown.

The reaction is traditionally described by Lowe–Thorneley scheme [10–12], which involves eight intermediates *E*_0_–*E*_8_, differing in the number of electrons and protons delivered to the enzyme. It has been shown that the enzyme needs to be loaded by three or four electrons and protons before N_2_ can bind. During the binding, H_2_ is released by a reductive elimination of two bridging hydride ions [1, 13]. Then, N_2_ is reduced and protonated, following either a distal or alternating mechanism [1]. In the former case, one N atom is first protonated and released as NH_3_ at the *E*_5_ level, before the second N atom is protonated. In the other mechanism, the protons are added alternatively to the two N atoms, so that HNNH and H_2_NNH_2_ are intermediates and the first NH_3_ product is not released until the *E*_7_ level. The alternating pathway is supported by the fact that nitrogenase can use hydrazine as a substrate and that hydrazine is released upon acid or base hydrolysis of the enzyme during turnover [1, 5, 11, 12]. It has also been shown that N_2_, N_2_H_2_, CH_3_N_2_H and N_2_H_4_ all react via a common intermediate [1, 14]. On the other hand, it has been shown that inorganic model complexes follow a sequential mechanism [15–19].

Many crystal structures of nitrogenase have been solved [8, 20–24]. They show that the main group of the nitrogenases has an active-site composed of an MoFe_7_S_9_C(homocitrate) cluster, connected to the protein by a histidine and a cysteine residue. However, there also exist alternative nitrogenases, in which the Mo ion is replaced by either vanadium or iron [25]. They are typically less effective than Mo-nitrogenase, forming more H_2_ by-product than shown in Eq. (1). The crystal structure of V-nitrogenase has been solved and it showed that the enzyme contains an extra subunit and that one sulphide ligand in the active-site cluster is replaced by a bidentate ligand, probably carboxylate [26].

**Fig. 1:**
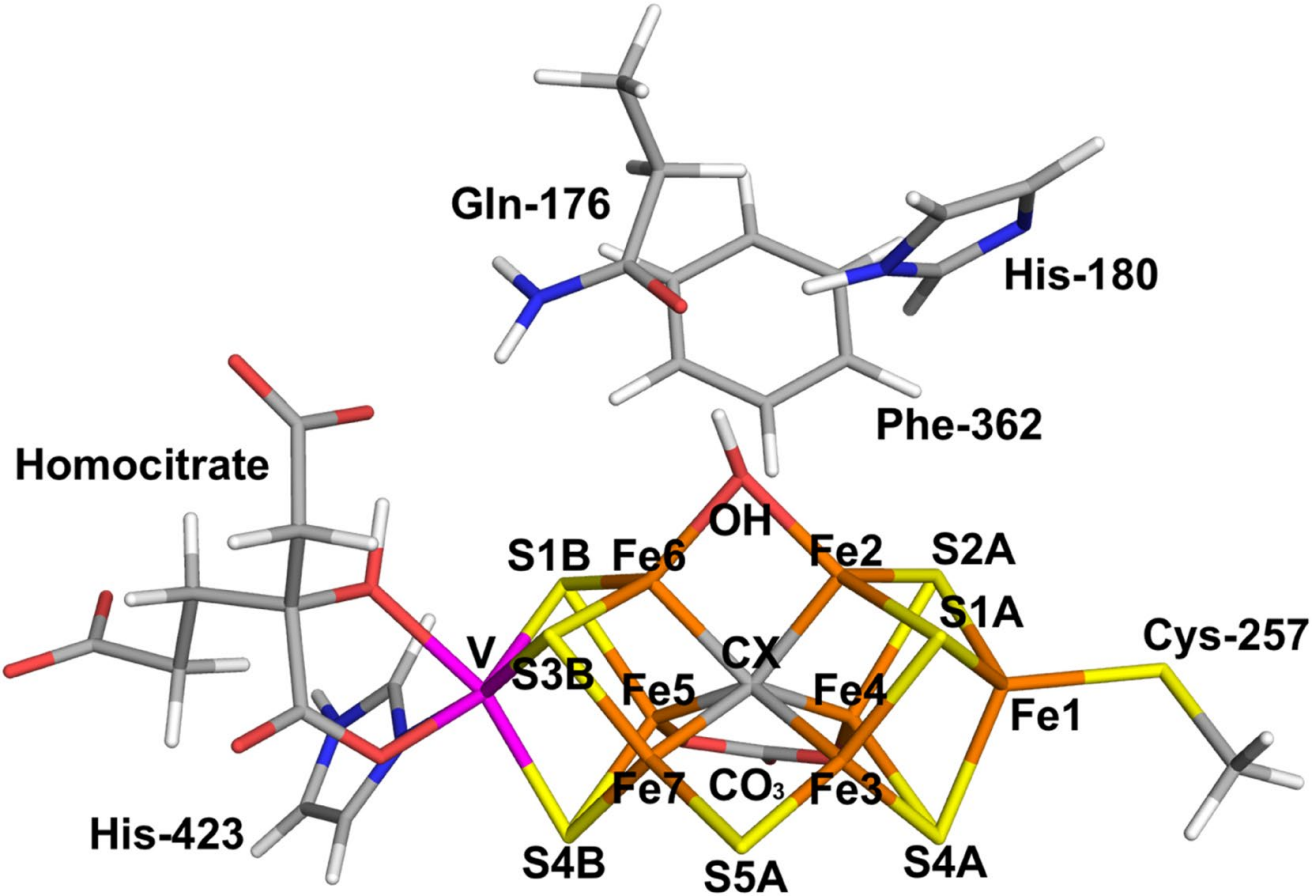
The nitrogenase FeV cluster with the S2B ion replaced by OH^−^ (bridging Fe_2_ and Fe_6_), showing the atom names (from the 6FEA crystal structure [27]) and the QM system employed in the quantum-refinement calculations

Recently, another crystal structure of V-nitrogenase was solved, using an enzyme obtained in a less reducing environment [27]. Excitingly, it was shown that one of the µ_2_ sulphide ligands (S2B) was replaced by a lighter atom, as is shown in Fig. 1 (replacement of S2B has also been observed in a CO-inhibited structure of Mo-nitrogenase [23]). Based on the analyses of the hydrogen-bond network and the electron density, it was suggested that this ligand is NH^2−^, representing the *E*_6_ reaction intermediate. Moreover, a new electron density was observed 7 Å from the FeV cluster, interpreted as a storage site for the released S2B ion. This site was formed by a change in the conformation of residue Gln-176, which rotated around the CA–CB bond so that it came closer to the active site and could accept hydrogen bonds from the substrate.

However, only a few months later, Bjornsson and coworkers presented a combined quantum mechanical and molecular mechanical (QM/MM) study of this structure [28]. They compared structures containing either NH^2−^ or OH^−^ replacing S2B and showed that the latter structures reproduced the Fe–S, Fe–Fe and hydrogen-bond distances in the original crystal structure better than NH^2−^. Therefore, they concluded that the crystal structure does not show any N_2_-derived reaction intermediate, but rather an OH^−^-bound state.

This is an interesting suggestion, but not fully conclusive (the original crystallographers still argue that the structure contains a reaction intermediate [29]), because the comparison is made to the coordinates of the original crystal structure and not directly to the crystallographic raw data. The coordinates are the result of involved cycles of model building and crystallographic refinement, involve a significant uncertainty (estimated to 0.026 Å for the atomic coordinates of present structure [27]), and are biased towards the atomic model, because the crystallographic phases cannot be measured in the experiment. A further important complication is that the crystal structure contains a significant amount of undissociated S2B ligand (< 5% estimated by the crystallographers [27], but we get 10–20% by occupancy refinement). This would favor a ligand with many electrons, but it also makes the original coordinates unreliable, because they do not represent a pure model, but rather a weighted mixture of two states.

Therefore, we here perform instead quantum refinement calculations on the V-nitrogenase crystal structure, to decide the nature of the bridging ligand. Quantum refinement is standard crystallographic refinement, in which the empirical restraints (which is employed in almost all crystal structures to ensure that bond lengths and angles make chemical sense) are replaced by more accurate quantum–mechanical calculations [30, 31]. This is especially important for metal sites, for which no accurate empirical restraints are available. This gives structures that are an ideal compromise between crystallography and quantum mechanics, and it allows us to decide what model fits the crystallographic raw data best by looking at standard crystallography quality measures, like the electron-density difference maps and the real-space *Z* scores [32].

## Methods

In standard crystallographic refinement, the structure is obtained as a compromise between the experimental data and a set of empirical restraints by minimising a target function of the form

2 $${E}_{\text{tot}}={{w}_{\text{A}}E}_{\text{Xray}}+{E}_{\text{MM}},$$

where ${E}_{\text{Xray}}$ is the experimental target function (describing how well the current model reproduces the experimental data; typically a maximum-likelihood function [33, 34]) and ${E}_{\text{MM}}$ is the empirical restraints (which in terms of computational chemistry is a molecular mechanics, MM, potential). ${w}_{\text{A}}$ is a weight factor that is needed because the two terms do not have the same units and it determines the relative importance of the terms. It is normally determined so that the MM and crystallographic forces are of an equal magnitude in a short molecular dynamics simulation of the system [34–36].

In quantum refinement, the empirical potential in Eq. (3) is replaced by more accurate quantum mechanical (QM) calculations for a small, but interesting part of the macromolecule, e.g. an enzyme active site (called system 1 in the following). This gives the target function

3 $${E}_{\text{Cqx}}={{{w}_{\text{MM}}(w}_{\text{A}}E}_{\text{Xray}}+{E}_{\text{MM}}-{E}_{\text{MM1}})+{E}_{{\text{QM1}}}$$

where ${E}_{\text{QM1}}$ is the QM energy of system 1, whereas ${E}_{\text{MM1}}$ is the MM energy of system 1 (needed to avoid double counting of this energy). ${w}_{\text{MM}}$ is a weighting factor needed because the empirical potential of crystallographic refinement software is normally based on a statistical analysis of high-resolution crystal structures [37], rather than on energetic consideration (as for the QM term). We have shown that quantum refinement can locally improve crystal structures [31], decide protonation state of metal-bound ligands [38–41], oxidation state of metal sites [42, 43] and protein ligands [41], detect photoreduction of metal ions [42, 44, 45] and decide what is really seen in crystal structures [44–46].

Quantum refinement calculations were performed with the ComQumX software [30], which is a combination of Turbomole [47] and the crystallography and NMR system (CNS) [48, 49], version 1.3. We also employed the recently developed extension of the method to systems with dual conformations in the QM system, ComQumX-2QM [50]. This approach employs the energy function

4 $$\begin{aligned} E_{{\text{Cqx-2QM}}} & = w_{{{\text{MM}}}} \left( {w_{{\text{A}}} E_{{{\text{Xray}}}} + E_{{{\text{MM}}}} - n_{{{\text{occ1}}}} E_{{{\text{MM11}}}} - n_{{{\text{occ2}}}} E_{{{\text{MM12}}}} } \right) \\ & \quad + n_{{{\text{occ1}}}} E_{{{\text{QM11}}}} + n_{{{\text{occ2}}}} E_{{{\text{QM12}}}} \\ \end{aligned},$$

where ${E}_{\text{Xray}}$ and ${E}_{\text{MM}}$ are the same as in Eqs. (1) and (2), but they now involve alternative conformations of atoms in the QM system. ${E}_{\text{QM11}}$ and ${E}_{\text{MM11}}$ are the QM and MM energies of the first conformation of the QM system (called system 11), which has the occupancy ${n}_{\text{occ1}}$. Likewise, ${E}_{\text{QM12}}$ and ${E}_{\text{MM12}}$ are the QM and MM energies of the second conformation of the QM system (called system 12), which has the occupancy ${n}_{\text{occ2}}$.

The quantum-refinement calculations were based on the recent crystal structure of V-nitrogenase with a putative N-derived reaction intermediate (1.2 Å resolution) [27]. Coordinates, occupancies, *B*-factors and structure factors were obtained from the 6FEA protein data bank files. From these files, we also obtained the space group, unit cell parameters, resolution limits, *R* factors and the test set used for the evaluation of the *R*_free_ factor.

The calculations were performed the same way as in our previous quantum-refinement studies for Mo-nitrogenase [39, 40, 50]: The full protein was used in all calculations, including all crystal-water molecules. For the protein, we used the standard CNS force field (protein_rep.param, water_rep.param and ion.param). However, CNS does not support anisotropic *B*-factors, so only isotropic *B*-factors were used. The empirical restraints for non-standard residues were downloaded from the Hetero-compound Information Centre Uppsala [51]. The *w*_A_ factor (determining the relative weight between the crystallographic data and the empirical potential) was the default value suggested by CNS, 0.1031 (but other values were tested in some calculations). The *w*_MM_ weight was set to 1/3 as in all our previous studies [30, 43]. For the crystallographic target function, we used the standard maximum-likelihood function using amplitudes (mlf) in CNS [33, 34]. After quantum refinement, anisotropic *B*-factor refinement was performed using *phenix.refine* [52]*.* The electron density maps were generated using *phenix.maps*.

The QM calculations were performed at the TPSS/def2-SV(P) level of theory [53, 54], but a few calculations were performed also with the TPSSh [55] and B3LYP methods [56–58], and with the def2-TZVP basis set [59]. The calculations were sped up by expanding the Coulomb interactions in an auxiliary basis set, the resolution-of-identity (RI) approximation [60, 61]. Empirical dispersion corrections were included with the DFT-D3 approach [62] and Becke–Johnson damping [63].

The quality of the models was compared using the real-space difference-density *Z* score (RSZD), calculated by EDSTATS (part of the CCP4 package [64]), which measures the local accuracy of the model [32]. The maximum of the absolute negative and positive RZSD value was calculated for the unknown ligand replacing S2B, as well as for Gln-176 and His-180. RSZD is typically less than 3.0 in absolute terms for a good model.

The FeV cluster was modelled by VFe_7_S_7_C(CO_3_)(homocitrate)(CH_3_S)(imidazole), where the two last groups are models of Cys-257 and His-423 (all mentioned residues are from the D subunit of the crystal structure). In addition, the putative N-derived ligand, as well as models of Gln-176, His-180 and Phe-362 were included in the QM calculations, as can be seen in Fig. 1 (in total 89–91 atoms, depending on the ligand replacing S2B). In a few calculations, we also included the nearby Lys-83, Arg-339 and Lys-361 residues (modelled by CH_3_NH_3_^+^ or CH_3_NHC(NH_2_)_2_^+^), to investigate the effect of partly neutralising the FeV cluster. We used the oxidation-state assignment ${\text{V}}^{\text{III}}{{\text{Fe}}}_{4}^{\text{II}}{{\text{Fe}}}_{3}^{\text{III}}$ of the metal ions in the resting *E*_0_ state of V-nitrogenase [28]. The homocitrate ligand was modelled in the singly protonated state with a proton shared between the hydroxyl group (which coordinates to V) and the O1 carboxylate atom. This protonation state was found to be the most stable one in Mo-nitrogenase [39, 65].

This gives a net charge of − 4 for the QM system in Fig. 1 for the resting state without the S2B ligand (i.e. with an empty coordination site) and therefore also with an NH_3_ ligand, which would represent the *E*_8_ state before product dissociation. The 6FEA crystal structure has been suggested to show the *E*_7_ state with an ${\text{NH}}_{2}^{ - }$ ligand or the *E*_6_ state with a NH^2−^ ligand (and we tested also the *E*_5_ state with a N^3−^ ligand). These are only formal charges on the ligands, because only the total charge of the QM system is defined. It is normally assumed that each pair of E_*n*_ and E_*n*+1_ states differ by the uptake of one electron and one proton, meaning that all E_*n*_ states have the same charge. Consequently, we used the same charge for the three models with N_2_-derived ligands (${\text{NH}}_{2}^{ - }$, NH^2−^ and N^3−^), − 4.

For the OH^−^-bound form, not so much information is available regarding the *E*_*n*_ state [66]. Direct OH^−^ binding to the resting *E*_0_ state with dissociated S2B would give a net charge of − 5. Other *E*_*n*_ states up to *E*_2_ are also possible, but the crystal structure does not show any evidence of protonation of the cluster (protonation of the sulphide or iron ions typically leads to significant changes in the Fe–S, Fe–C and Fe–Fe bond lengths by 0.1–0.6 Å [40]). Therefore, we used a net charge of − 5 for the OH^−^ complex (and − 6 for O^2−^, which we also tested). However, for all ligands, we tested also a few additional charge states, presented in Table S1 in the supporting information. Bjornsson and coworkers studied the same charge state with OH^−^ and NH^2−^, as well as a two-electron more oxidised state for OH^−^ and a two-electron more reduced state for NH^2−^ [28].

In QM calculations, the spin state should also be defined. The crystallographers reported a 38/62% mixture of para- and diamagnetic states of V-nitrogenase as isolated for the crystal (interpreted as a mixture of the *E*_6_ and *E*_7_ states) and assumes *S* = 3/2 state for all even-numbered *E*_*n*_ intermediates [27]. Bjornsson and coworkers also used the *S* = 3/2 state in all their calculations [28]. Previous studies have shown that it is very hard to decide the spin state of the active-site cluster from the QM energies [40]. Therefore, we tested several different spin states for each complex around the *S* = 3/2 or 2 state for *E*_*n*_ states with even and odd *n*, respectively. Fortunately, it turned out that the various spin states gave very similar structures and therefore RSZD scores (cf. Table S1).

The electronic structure of all QM calculations was obtained with the broken-symmetry (BS) approach [67]. Each of the seven Fe ions were modelled in the high-spin state, with either a surplus of α (four Fe ions) or β (three Fe ions) spin. We employed the broken-symmetry BS7-235 state with β spin on Fe_2_, Fe_3_ and Fe_5_ for all calculations. This is the best BS state for the resting state of Mo-nitrogenase and also for several other *E*_*n*_ states [40, 67, 68] and this state was also used in the previous study by Bjornsson and coworkers [28]. This state was obtained using the fragment approach by Szilagyi and Winslow [69] or by swapping the coordinates of the Fe ions [70].

## Result and discussion

### Quantum refinement of V-nitrogenase

We performed quantum refinement of the 6FEA crystal structure of V-nitrogenase [27], with a light ligand replacing S2B. Quantum refinement is a normal crystallographic refinement in which the empirical restraints, employed to give reasonable bond lengths and angles, are replaced by QM calculations for a small, but interesting part of the structure [30, 31]. We employed the QM system shown in Fig. 1. We performed quantum refinement for a number of structural interpretations of the electron density, differing in the nature of the N/O ligand (N^3−^, NH^2−^, ${\text{NH}}_{2}^{ - }$, OH^−^ or O^2−^), the protonation state of His-180 (with a proton on NE2 or ND1, called the HIE or HID states), the net charge of the QM system (i.e. the oxidation state) and the spin state of the FeV cluster. For the HIE structure, His-180 may donate a hydrogen bond to the ligand, whereas in the HID structure, it can instead accept a hydrogen bond. Therefore, only the HIE structure was tried for N^3−^ and O^2−^, which can only accept hydrogen bonds, and only the HID structure was used for ${\text{NH}}_{2}^{ - }$, which can only donate hydrogen bonds, whereas both states were tested for NH^2−^ and OH^−^. However, in the resulting structures it turned out that the ${\text{NH}}_{2}^{ - }$ ligand did not form any hydrogen bonds to Hid-180, so we tried also the HIE state for this ligand. In fact, all quantum-refined structures show a hydrogen bond between NE2 of His-180 and OE1 of Gln-176, rather than any hydrogen bond between the ligand and His-180. As discussed in “Methods”, we used a net charge of − 4 for the N_2_-derived ligands, which would correspond to the *E*_5_, *E*_6_ and *E*_7_ states for N^3−^, NH^2−^ and ${\text{NH}}_{2}^{ - }$, respectively. For OH^−^ and O^2−^, we used a charge of − 5 and − 6, respectively, corresponding to the binding of the ligands to the resting *E*_0_ state. Other charge states were also tested and those results are given in Table S1 in the Supplementary Material.

The results are presented in Table 1. We used the RSZD score [32] for the ligand, Gln-176 and His-180 (the two closest residues, forming hydrogen bonds to the ligand), to decide which structure fits the crystallographic raw data best. It can be seen (especially in Table S1) that there were only minimal differences in RSZD scores between different spin states of the cluster, reflecting that they gave essentially identical geometries. Moreover, the RSZD score for His-195 was similar for all tested models, 2.0–2.2. On the other hand, there were extensive differences in the RSZD value for the ligand. An OH^−^ ligand gave the lowest RSZD, 9.7 with HIE and 10.0 with HID, whereas NH_2_^2−^ gave 12.4 and the other ligands gave much worse results, 16–22. The RSZD score of Gln-191 showed an intermediate variation, ranging from 8.9 to 9.2 for N^3−^, NH^2−^ and OH^−^ with HID to 11.3 for O^2−^. Summing the three RSZD scores clearly shows that OH^−^ fits the crystal structure best, with a slight preference for the HID structure (21–22, compared to 25–33 for the other ligands). However, in energy terms, the HIE conformation is 104 kJ/mol more stable than the HID conformation (for the isolated QM system, cf. Table S1).

**Table 1:** RSZD scores for the ligand, Gln-176 and His-180 (sum is the sum of these three values) from the quantum-refinement calculations for the 6FEA crystal structure with different interpretations of the ligand replacing S2B (*X*), protonation states of His-180 and spin states (*S*)

*X*	Charge	His-180	*S*	RSZD score			
				Gln	His	*X*	Sum
N^3−^	− 4	HIE	2	9.1	2.0	21.9	33.0
			3	8.9	2.0	22.1	33.0
NH^2−^	− 4	HID	3/2	9.2	2.0	17.7	28.9
		HIE	3/2	9.7	2.0	17.8	29.5
${\text{NH}}_{2}^{ - }$	− 4	HID	2	10.8	2.1	12.4	25.3
OH^−^	− 5	HID	3/2	9.1	2.2	10.0	21.3
		HIE	3/2	10.3	2.2	9.7	22.2
O^2−^	− 6	HIE	3/2	11.3	2.1	16.2	29.6
OH^−^/S2B		HIE		2.8/2.4	2.0	1.8/1.7	7.1

The last line shows the results of a structure with both OH^−^ (83% occupancy) and S2B (17% occupancy) and two conformations of Gln-176 (89% occupancy of the flipped conformation and 11% occupancy of the non-flipped conformation; cf. Fig. 3d)

The structures with other oxidation states in Table S1 show similar trends, but with larger variations: OH^−^ always give a smaller RSZD score for the ligand (6.6–11.2) than the other ligands (11.6–22.1) with the trend OH^−^ < ${\text{NH}}_{2}^{ - }$ < O^2−^ < NH^2−^ < N^3−^. The RSZD score of His-180 is still minimal (1.9–2.3; but 2.8 in one case). However, the RSZD score of Gln-176 shows a larger variation, with the lowest values (6.2–6.3) for OH^−^ with HID in the − 2 charge state. The least negatively charged states (− 3) also give low scores for NH^2−^ and O^2−^ (8.0–8.4), whereas the most negative states give the highest RSZD scores, especially for the N-derived ligands (12.3–13.1). Consequently, the sum of the RSZD scores still points to OH^−^ as the best ligand (17.7–22.2, compared to 24–35). For all systems, the HIE conformation is more stable than the HID state in energy terms. The different spin state always give similar results and they are also close in energy.

**Fig. 2:**
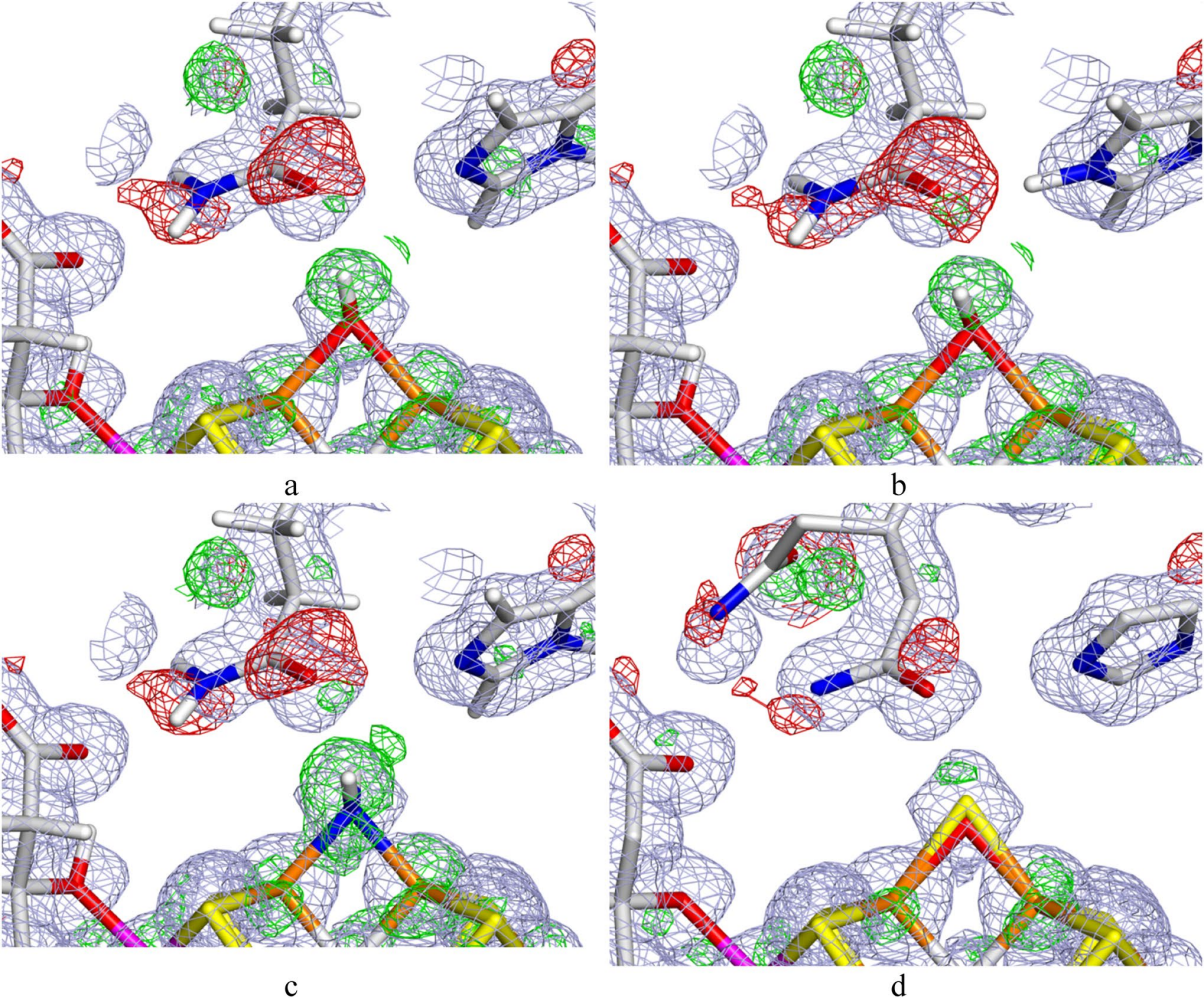
Electron-density maps of the best quantum-refined models of the 6FEA crystal structure: **a** OH^−^–HID, **b** OH^−^–HIE and **c** ${\text{NH}}_{2}^{ - }$–HID. **d** Shows a structure with both OH^−^ (83% occupancy) and S2B (17% occupancy) and two conformations of Gln-176 (89% occupancy of the flipped conformation, employed in the other structures, and 11% occupancy of the non-flipped conformation). The 2*mF*_o_ − *DF*_c_ maps are contoured at 1.0 *σ* (blue) and the *mF*_o_ − *DF*_c_ maps are contoured at + 3.0 σ (green) and − 3.0 *σ* (red)

Figure 2 shows the electron-density maps of the three best structures in Table 1. It can be seen that they are quite similar for the OH^−^ structures with HIE and HID, although the negative difference density around Gln-176 is somewhat smaller for the latter. Moreover, it is clear that the positive difference density is smaller around the OH^−^ ligand than around the ${\text{NH}}_{2}^{ - }$ ligand (compare Fig. 2a and b with c), as was also indicated by the RSZD score.

Still, the large positive difference densities around all ligands indicate that the S2B ligand has not fully dissociated (a positive difference density indicates that there should be a heavier atom at this position and it is also situated at a longer distance from the Fe_2_ and Fe_6_ ions, which reflects that sulphur gives longer metal bond lengths than the lighter atoms). This is also supported by the negative difference density around Gln-176, which indicates that this residue does not have a full occupancy in this flipped conformation. This is also the reason why the RSZD scores are so high for these two residues (RSZD should be less than 3 for an acceptable fit). The crystallographers also reported a small amount of the resting state (< 5%) in the structure [27].

To test this hypothesis, we set up a standard crystallographic refinement with two conformations for Gln-176 (the flipped conformation, used for all the other structures, and a non-flipped conformation) and both a sulphur ion and a hydroxide ion in the S2B position with fractional occupancies and then refined only the occupancies of these two groups, keeping the coordinates fixed. This led to occupancies of 17% for S2B and 83% for OH^−^. Likewise, the occupancies for Gln-176 became 89% for the flipped conformation and 11% for the original conformation. The corresponding electron-density maps are shown in Fig. 2d. It can be seen that they are strongly improved, even if there is still some negative density close to the OH/S2B ion and both positive and negative densities around the two conformations of Gln-176. In fact, the RSZD scores have decreased to 1.7–2.8 for these two groups, indicating an acceptable fit (shown in the last line of Table 1).

### Quantum refinement with dual conformations in the QM system

This observation opens for the possibility that the preference of the OH^−^ ligand simply reflects that oxygen contains more electrons than nitrogen and therefore provides a better fit the experimental electron density, because the latter involves a significant amount of the heavier S2B ligand still bound to the FeV cluster.

To check this possibility, we repeated the quantum-refinement calculations with the recently developed extension allowing for dual conformations within the QM system [50]. In these calculations, we let the complete QM system (shown in Fig. 1, i.e. the FeV cluster, Gln-176, His-180 and Phe-362) to have two distinct conformations. In the first conformation (86% occupancy, i.e. the average of the occupancies obtained for S2B and Gln-176 in the occupancy refinement for the structure in Fig. 2d), the unknown ligand binds to the cluster, replacing S2B, which has moved to the storage site, and Gln-176 is in the flipped conformation observed in the crystal structure [27]. In the second conformation (14% occupancy), S2B remains bound to the FeV cluster and Gln-176 is in the non-flipped conformation, observed in all previous crystal structures [22]. It was assumed that the latter structure is in the *E*_0_ resting state with a total charge of − 6, *S* = 3/2 and His-180 in the HIE state (because His-180 donates a hydrogen bond to S2B in that conformation) [39].

We tested the same five different interpretations of the unknown ligand as in the previous section (N^3−^, NH^2−^, ${\text{NH}}_{2}^{ - }$, OH^−^ or O^2−^) and either the HID or HIE state of His-180, in total eight different structures (with the preferred net charge and spin state from Table 1). The results are collected in Table 2. It can be seen that the results are significantly better, but show the same trends as in Table 1. The second conformation is the same in all systems and therefore shows only minimal variation of the RSZD scores between the various systems (0.0–0.1 for His-180, 5.7–6.0 for Gln-176, but 0.3–7.9 for S2B, reflecting that it overlaps strongly with the unknown ligand in the first conformation).

**Table 2:** Results of the ComQumX-2QM calculations for the 6FEA crystal structure

*X*	*w*_A_	*n*_occ_	*q*	His	*S*	RSZD AC1			RSZD AC2			RSZD	∆*E*_QM_	
						Gln	His	*X*	Gln	His	S2B	Sum	AC1	AC2
N^3−^	0.1	14	− 4	HIE	2	6.6	1.0	5.1	5.9	0.0	7.9	26.5	157	10
NH^2−^			− 4	HID	3/2	5.8	1.3	3.2	5.8	0.0	5.8	21.9	181	10
			− 4	HIE		7.3	1.1	3.5	5.9	0.0	5.6	23.4	145	10
${\text{NH}}_{2}^{ - }$			− 4	HID	2	8.5	1.3	5.8	5.7	0.0	2.2	23.5	175	10
			− 4	HIE		9.1	1.1	5.8	5.7	0.1	2.2	24.0	143	10
OH^−^			− 5	HID	3/2	7.1	1.3	0.9	5.8	0.0	0.8	15.9	165	10
			− 5	HIE		9.8	1.2	0.9	6.0	0.1	0.3	18.3	124	10
O^2−^			− 6	HIE	3/2	11.5	1.2	1.0	6.0	0.0	2.8	22.5	148	10
OH^−^	0.1	14	− 5	HID^a^	3/2	10.0	1.5	1.2	5.8	0.1	0.5	19.1	198	10
NH^2−^	0.1	11	− 4	HID	3/2	6.9	1.4	4.3	4.2	0.0	6.6	23.4	182	8
		14				5.8	1.3	3.2	5.8	0.0	5.8	21.9	181	10
		17				5.2	1.2	2.9	7.1	0.0	4.0	20.4	180	12
OH^−^		11	− 5	HIE	3/2	10.8	1.3	1.3	4.4	0.0	0.5	18.3	124	8
		14				9.8	1.2	0.9	6.0	0.1	0.3	18.3	124	10
		17				8.7	1.1	0.6	7.3	0.0	0.4	18.1	124	12
OH^−^	0.1	11	− 5	HIE	3/2	10.8	1.3	1.3	4.4	0.0	0.5	18.3	124	8
	0.01					24.2	10.6	2.9	4.6	4.3	1.1	47.7	22	0
	0.001					25.0	17.4	4.9	5.0	5.9	1.8	60.0	3	0
	0					25.2	18.5	4.0	4.9	4.2	1.8	58.6	0	0

Two conformations were used for the QM system. The first (AC1 with occupancy 100 − *n*_occ_) represents the conformation reported in the crystal structure, involving an unknown ligand (*X*) replacing S2B and Gln-176 in the flipped conformation (with the cluster charge, *q*, spin state, *S*, and His-180 state shown in the table). S2B is in the storage site, 7 Å from the FeV cluster. The other conformation (AC2 with occupancy *n*_occ_) represents a normal *E*_0_ resting state (i.e. with a cluster charge of − 6, *S* = 3/2 and His-180 in the HIE state) with S2B bound to the cluster and Gln-176 in a non-flipped conformation. The RSZD scores are calculated for the ligand, Gln-176 and His-180 for the two alternative conformations (sum is the sum of these six values). The last two columns represent the strain energies of the two QM systems in kJ/mol

^a^With Gln-176 rotated so that it interacts with the OH^–^ ligand with the side chain –NH_2_ group

For the first conformation, the variation in the RSZD scores is somewhat larger, although the His-180 residue gives 1.0–1.3 for all systems. Clearly, the lowest RSZD scores for the unknown ligand are obtained with OH^−^ and O^2−^ 0.9–1.0, compared to 3.2–5.8 for the other ligands. On the other hand, Gln-176 gives the best results for NH^2−^, N^3−^ and OH^−^, especially with HID (5.8–7.3). The other structures give 8.5–11.5. Summing the RSZD scores of all three residues in the two conformations (column sum in Table 2) shows that the best result is obtained for the two OH^−^ structures (15.9 and 18.3). The other structures have sums of 22–27.

We also tested an OH^−^–HID structure with Gln-176 rotated around the CG–CD bond (so that OE1 and NE2 changes positions). Then, the protons on NE2 can form hydrogen bonds to ND1 of His-180 and to the ligand. However, this gave slightly worse results (the sum of the RSZD scores is 19.1).

**Fig. 3:**
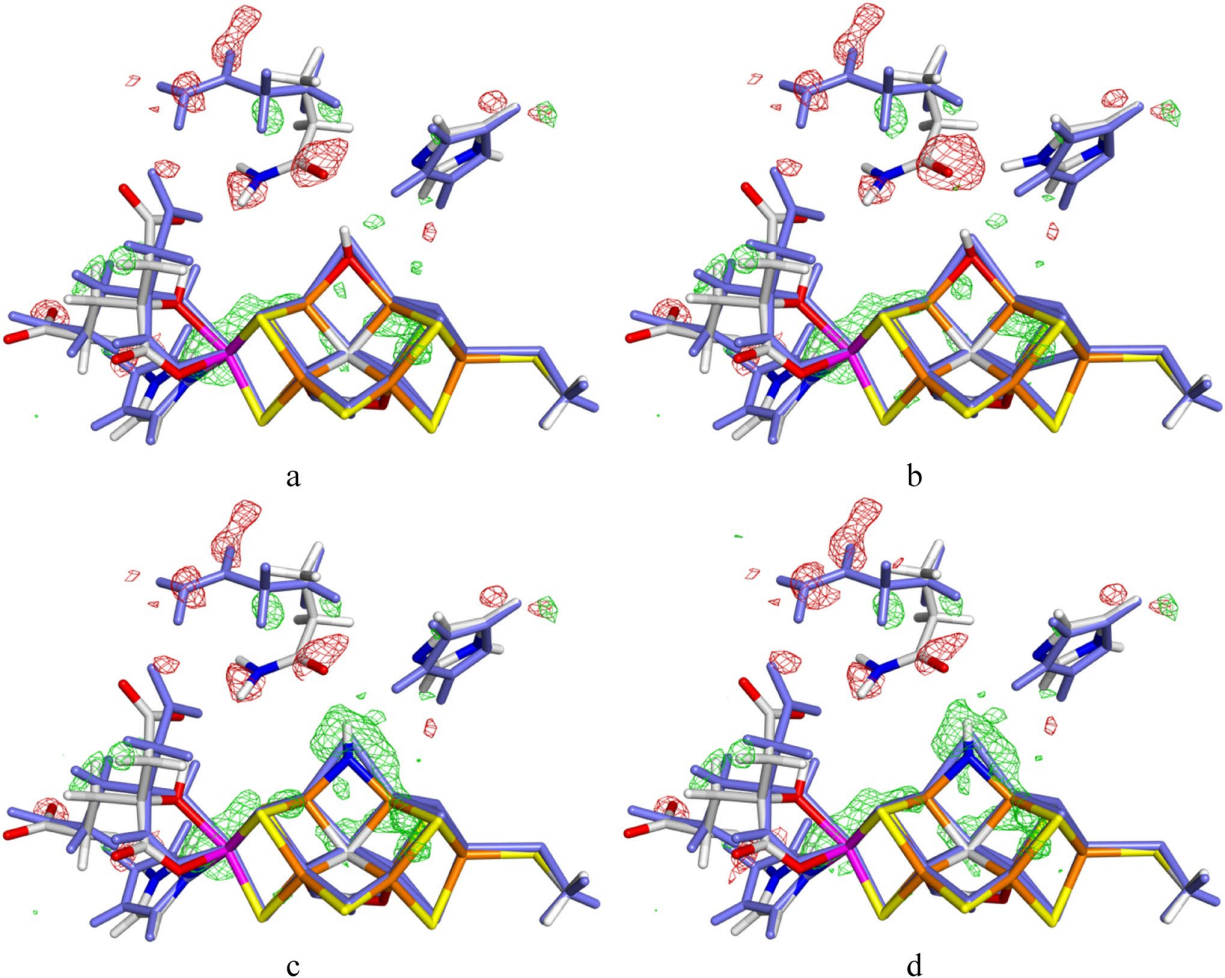
Electron-density maps of the best ComQumX-2QM quantum-refined models of the 6FEA crystal structure: **a** OH^−^–HID, **b** OH^−^–HIE and **c** NH^2−^–HID. Two conformations were used for the QM system. The first (with 86% occupancy, shown with atomic colours) represents the conformation reported in the crystal structure, involving an unknown ligand replacing S2B to the storage site and Gln-176 in the flipped conformation. The other conformation (14% occupancy, shown in pale cyan) represents a normal E_0_ resting state with S2B bound to the cluster and Gln-176 in a non-flipped conformation. **d** Shows the NH^2−^–HID structure obtained with occupancies of 83 and 17%, respectively. The 2*mF*_o_ − *DF*_c_ difference maps are contoured at + 3.0 *σ* (green) and − 3.0 *σ* (red)

The variation in the RSZD scores is also reflected in the electron-density difference maps, which are shown for the three best structures in Fig. 3. It can be seen that the OH^−^ structures provide a much better description of the unknown ligand than NH^2−^ (a large positive difference density around the latter ligand). On the other hand, the OH^−^–HIE structure gives a somewhat worse result around the OE1 atom of the flipped conformation of Gln-176, whereas it is slightly better around the NE2 atom. It is notable that in both cases, there are positive electron density around the OE1 and NE2 atoms of Gln-176 in both conformations, which may indicate that it actually attains additional conformations.

We also observed that if the displaced S2B ion in the storage site is modelled with only partial occupancy (86%), a large negative density is obtained around it (RSZD ≈ 25). It required a full occupancy and a rather large *B* factor (~ 15) to obtain a proper model (RSZD = 1.2–1.9). This indicates that there is not a direct relation between the occupancy of S2B at the FeV cluster or in the storage site.

To get some further indication of which of the structures fit the crystallographic raw data best, we have also calculated the strain energies (∆*E*_QM_) of the two QM systems [38, 46, 50], i.e. the difference in the QM energy when optimised in the crystal structure and when optimised without the crystallographic data (i.e. with *w*_A_ = 0). These energies are shown in the last two columns of Table 2. It can be seen that the strain energy of the second conformation (i.e. *E*_0_ with S2B bound to the cluster) is the same in all calculations, 10 kJ/mol. However, for the first conformation, there are large differences, depending on the ligand. The lowest strain energies are obtained for the OH^−^ ligand with HIE, 124 kJ/mol. In fact, all HIE structures have lower strain energies, 124–157 kJ/mol, than the HID structures, 165–181 kJ/mol. However, also among the HID structures, OH^−^ gives the lowest strain. The HIE structures also always give a lower QM energy than the corresponding HID structure, by 92–104 kJ/mol (results not shown), showing that the HIE structures is more stable. Consequently, we tend to prefer the HIE structures, especially as the crystal structure shows a clear hydrogen between His-180 and Gln-176 (the slightly worse RSZD score for the OH^−^–HIE structure may reflect a small overestimation of the strength of this hydrogen bond with the TPSS functional and the high negative charge of the QM systems).

The large positive difference density around NH^2−^ in Fig. 3c indicates that the structure might be improved by using a higher occupation number for the second conformation. Therefore, we performed calculations of the NH^2−^–HID structure with occupancies for the second conformation of 11 and 17% also (the values obtained for Gln-176 and S2B in the Phenix occupancy optimisation; and 89% or 83% occupancy for the first conformation). The results in Table 2 and in Fig. 3d show that there is a slight improvement of RSZD scores and the difference density around NH^2−^ as the occupancy of the second conformation is increased, but the structure is still much worse than for OH^−^ (the RSZD of NH^2−^ is 2.9, compared to 0.9 for OH^−^). It can also be seen that the strain energy of the first conformation decreases slightly (182–180 kJ/mol, whereas that of the second conformation increases slightly with the occupancy (from 8 to 12 kJ/mol). This is expected [50]: when the occupancy is increased, the restraint towards the crystal structure is increased. This is also the reason why the strain energy is much larger for the first QM system than for the second.

For OH^−^–HIE, the RSZD scores of the ligand shows a slight decrease with the occupancy (Table 2). However, Gln-176 shows larger changes, but the first conformation is improved when the occupancy is increased, whereas the second conformation is deteriorated, so that the sum of the RSZD scores hardly changes with the occupancy.

The strain energy of the first conformation (124–181 kJ/mol) may be considered to be somewhat large, compared to other systems [50]. Therefore, we performed a few calculations also with other values of the *w*_A_ weight factor. All previous calculations used the default weight calculated by CNS to 0.1. Reducing *w*_A_ to 0.01 strongly reduced both strain energies, to 22 and 0 kJ/mol, respectively (also shown in Table 2). However, the fit to the crystal structure was also much deteriorated (the sum of the RSZD scores increased to 48). Therefore, we decided to accept the results at *w*_A_ = 0.1

Finally, in Table 3, we show how the various calculations reproduce the distances in the original crystal structure [27]. Results are shown only for the first conformations (with the unknown ligand and the flipped conformation of Gln-176, because the second conformation is the same for all systems and therefore shows very small differences between the various systems. It can be seen that the two quantum refinements with OH^−^ give the lowest mean absolute deviation (MAD) for the 15 short (< 3 Å) metal–metal distances from the original crystal structure, 0.005–0.006 Å. However, the MAD is not much larger for the other ligands, 0.007–0.010 Å, because all refinements employ the crystallographic raw data. The maximum deviation shows a larger variation, 0.02 Å for OH^−^ and ${\text{NH}}_{2}^{ - }$ and 0.04–0.06 Å for the other ligands. The deviations are appreciably larger for the 34 short metal–ligand distances (< 2.5 Å), but the trends are similar: The MAD is 0.02 Å for ${\text{NH}}_{2}^{ - }$ and OH^−^, but 0.03 Å for the other three ligands and the maximum errors are 0.08–0.10 Å for ${\text{NH}}_{2}^{ - }$ and OH^−^, but 0.21–0.32 Å for the others. These differences should be put in relation to the positional uncertainty of the atoms in the structure, which according to Cruickshank’s diffraction precision index is 0.026 Å [27].

**Table 3:** Geometry results of the ComQumX-2QM calculations for the 6FEA crystal structure

	His	*w*_A_	*n*_occ_	Metal–metal		Metal–S/O/C	
				MAD	Max	MAD	Max
N^3−^	HIE	0.1	14	0.010	0.059	0.032	0.321
NH^2−^	HID			0.009	0.043	0.029	0.218
	HIE			0.009	0.040	0.029	0.218
${\text{NH}}_{2}^{ - }$	HID			0.007	0.020	0.017	0.088
	HIE			0.007	0.018	0.018	0.084
OH^−^	HID			0.005	0.018	0.020	0.109
	HIE			0.006	0.016	0.019	0.091
O^2−^	HIE			0.009	0.044	0.027	0.210
OH^−^	HID^a^			0.006	0.013	0.019	0.088
NH^2−^	HID	0.1	11	0.009	0.041	0.029	0.213
			14	0.009	0.043	0.029	0.218
			17	0.010	0.046	0.029	0.224
OH^−^	HIE	0.1	11	0.006	0.017	0.019	0.086
			14	0.006	0.016	0.019	0.091
			17	0.006	0.016	0.020	0.096
OH^−^	HIE	0.1	11	0.006	0.017	0.019	0.086
		0.01		0.027	0.062	0.030	0.070
		0.001		0.050	0.111	0.035	0.081
		0		0.056	0.118	0.036	0.084

Two conformations were used for the QM system. The calculations are the same as in Table 2. Listed are the mean absolute deviations (MAD) and maximum deviations from the starting crystal structure in the metal–metal and metal–ligand distances

^a^With Gln-176 rotated so that it interacts with the OH^−^ ligand with the side chain –NH_2_ group

Interestingly, the maximum errors are always observed for the Fe_2_–Fe_6_ and Fe_2_–*X* or Fe_6_–*X* distances (where *X* is the unknown ligand) and the quantum-refined distances are always shorter than in the original crystal structure. This shows that the original crystal structure is significantly affected by the partly remaining S2B ligand, which makes the distances too long (especially the Fe2/6–*X* distances). This illustrates the problem of basing the judgement of what structure fits the crystallographic data best on distances from the original crystal structures, as done by Bjornsson and coworkers [28]. The current approach of re-refining the structures, taking into account the dual conformations in the active site and using RSZD scores, electron-density difference maps and strain energies is much more accurate.

### Sensitivity of the results

Quantum refinement is a combination of crystallographic refinement and QM calculations. A natural question is then how much the results depend on the QM method and model. We have already discussed how the results depend on the charge and spin state used for the FeV cluster (Table S1). In particular, we pointed out that the conclusion that OH^−^ fits the crystal structure best remained even if the charge of the cluster was varied by four units. Structures with the same ligand, but different charge state, are closely similar. For example, the coordinates of OH^−^–HIE structure with *S* = 2 and a net charge of − 2 or − 6 differ by only 0.05 Å on average, with a largest movement of 0.14 Å for the OE1 atom of Gln-176. The MADs of the metal–metal and metal–ligand distances are 0.009 and 0.023 Å, respectively.

In Table 4, we present results for the ComQumX-2QM OH^−^–HIE structure with some variations in the theoretical method. First, we have used two different DFT methods. TPSSh was used by Bjornsson and coworkers [28] and has been shown to give structures of a similar quality as TPSS for the active-site FeMo cluster in the resting state of Mo-nitrogenase. From Table 4, it can be seen that TPSSh gives RSZD scores similar to those for TPSS: The RSZD is slightly lower for the first conformation of Gln-176, but higher for the OH^−^ group. The sum of the RSZD scores decreases from 18.3 to 17.9. On the other hand, the strain energies are larger, 143 and 21 kJ/mol, compared to 124 and 10 kJ/mol for TPSS. However, the change in the coordinates is minimal, 0.01 Å for the first conformation and the MADs for the metal–metal and metal–ligand distances are only 0.003 and 0.005 Å. For the other conformation, the differences are appreciably larger, 0.05 Å for coordinates and 0.066 and 0.027 Å for the two sets of distances. The reason for this is the low occupancy of the second conformation, which reduces the restraint towards the crystallographic data.

**Table 4:** Sensitivity of the results to variations in the method. ComQumX-2QM calculations were performed for the OH^−^–HIE structure with *w*_A_ = 0.1 and *n*_occ_ = 0.14 and the same two alternative conformations as in Table 2

Method	RSZD							∆*E*_QM_		MAD (Å)					
	AC1			AC2				kJ/mol		AC1			AC2		
	Gln	His	OH	Gln	His	S	Sum	AC1	AC2	M–M	M–L	Coord	M–M	M–L	Coord
Standard QM model															
TPSS	9.8	1.2	0.9	6.0	0.1	0.3	18.3	123.9	9.9						
TPSSh	9.0	1.2	1.1	6.1	0.1	0.4	17.9	143.3	21.2	0.003	0.005	0.010	0.066	0.027	0.046
B3LYP	9.0	1.2	1.1	5.7	0.1	0.4	17.5	223.5	35.4	0.004	0.010	0.015	0.136	0.050	0.095
QM system enlarged with Lys-83, Arg-339 and Lys-361															
TPSS	5.6	1.0	0.5	4.7	0.0	0.9	12.7	208.1	20.4	0.003	0.010		0.026	0.025	
TPSSh	5.2	1.1	0.3	4.8	0.0	0.8	12.2	223.4	20.1	0.002	0.004	0.010	0.064	0.026	0.041
TZVP	5.5	1.0	0.6	4.8	0.0	1.3	13.2	200.1	17.6	0.004	0.010	0.013	0.066	0.045	0.052

The three first lines were obtained with the standard QM system, shown in Fig. 1, using charges of − 5 and − 6 for the two alternative conformations (with OH^–^ and S2B, respectively). In the last three lines, the QM system was enlarged by side chain models of Lys-83, Arg-339 and Lys-361, giving charges of − 2 and − 3. The MAD columns show the mean absolute deviation of the metal–metal and metal–ligand distances, as well as all coordinates, compared to the TPSS structure with the same model, or compared to the TPSS structure with the standard model (TPSS with the enlarged QM system). The calculation in the last line was performed with the TPSSh method with the def2-TZVP metal ions, sulphur, the central carbide ion and OH^−^ and def2-SV(P) on the other atoms

If we instead use the B3LYP method, which is known to give a quite poor structure of the resting state of the FeMo cluster [71], the results are similar: The sum of the RSZD scores is 17.5. The MADs compared to the TPSS structure are somewhat larger than for TPSSh for the first conformation, 0.004–0.015 Å, and they have doubled for the second conformation, 0.005–0.14 Å. Again, this reflects the stronger restraints to the crystal structure for the first conformation.

For both methods, the differences are much lower in the quantum-refined structures than if the QM systems are optimised without any restraints to the crystallographic data (i.e. with *w*_A_ = 0, implicating a pure QM/MM method, with the CNS force field). For example, the MADs for the metal–metal and metal–ligand distances for first conformation then become 0.10 and 0.04 Å with TPSSh and 0.19 and 0.06 Å for B3LYP. This shows that quantum refinement is much less sensitive to the DFT method than QM/MM calculations, because the structure is mainly determined by the crystallographic data.

Next, we tested to enlarge the QM system with models also of the side chains of Lys-83, Arg-339 and Lys-361. These groups were included in the calculations of Bjornsson et al. [28] and they partly compensate the negative charge of the FeV cluster. From the second part of Table 4, it can be seen that this improved the RSZD scores considerably: The sum was reduced from 18.3 to of 12.7, using the TPSS method. It is especially the RSZD score of Gln-176 that was improved in both conformations, viz. to 4.7–5.6. However, the metal–metal and metal–ligand distances of the cluster do not change much: The MADs are 0.003 and 0.010 Å for the first conformation and 0.026 and 0.025 Å for the second. On the other hand, the strain energies increased to 208 and 20 kJ/mol. This is quite expected, because the QM system has been enlarged and three positively charged groups have been added—strain energies are comparable only for QM systems of the same size.

Finally, we changed the DFT method to TPSSh and also enhanced the basis set to def2-TZVP on the metal ions, sulphur, the central carbide ion and OH^−^ (i.e. the basis set used by Bjornsson and coworkers [28]), using also the large QM system. From the results in Table 4, it can be seen that the effect of the change in the functional is very similar to that for the smaller QM system, whereas the effect of the change in the basis set is slightly larger, although the sum of the RSZD scores deteriorates slightly (to 13.2). Consequently, we can conclude that for this high-resolution structure, the QM method and basis set have quite restricted effect on the final structure. The choice of the QM model is slightly more important and it seems to be favourable to compensate the negative charge of the QM model as much as possible.

Finally, and most importantly, it should be pointed out this method dependence is not a disadvantage introduced by quantum refinement. Standard crystallographic refinements also depend on the empirical restraints (*E*_MM_ in Eq. 1). This has become such an integrated part of crystallography that it is normally not discussed, but for low- and medium-resolution protein structures it determines the details of the structure, i.e. the bond lengths and angles. Therefore, the final structure will strongly depend on these restraints. Fortunately, they are accurate for standard parts of the protein, i.e. the amino acids, because it is based on statistical analysis of many high-resolution crystal structures. However, for cofactors, substrates and inhibitors less prior information is available and consequently, the restraints are much less accurate. The same applies to metal sites and for these, it is also very hard to construct reliable MM force fields [72] and they depend strongly on the charge on the metal and the nature of all ligands. In practice, they are often modelled simply by a Lennard–Jones potential. Different crystallography softwares typically have different approaches to treat such hetero-compounds and it is often left to the crystallographer to decide what potential to use or to construct the potential himself. Consequently, the result will depend on the software and how the potential was obtained, although this is seldom discussed. Quantum refinement partly solves these problems by employing QM calculations, which are appreciably more accurate than MM calculations. The fact that the results depend on the charge and protonation state used for the calculations illustrates that the method is so accurate that these details matter. Quantum refinement partly shifts the focus towards these problems, which normally are overlooked in standard crystallography, but they are not caused or introduced by the method.

## Conclusions

We have performed a detailed investigation of the recent crystal structure of V-nitrogenase with a putative N_2_-derived reaction intermediate [27]. The crystallographers suggested that it shows the *E*_6_ (NH^−^) or *E*_7_ (${\text{NH}}_{2}^{ - }$) reaction intermediates in the Lowe–Thorneley scheme [10–12]. This would be quite sensational as it would settle some important aspects of the highly controversial [1, 9] reaction mechanism, viz. that one of the µ_2_ sulphide ligands (S2B) dissociates to form a binding site of the substrate between the Fe_2_ and Fe_6_ ions, and that it is stored in a binding site, close to the FeV cluster. On the other hand, it is quite unexpected that a crystal structure should show a reaction intermediate, because they are normally very short-lived.

We have used quantum refinement to deduce what ligand fits the crystal structure best. This approach can be seen as standard crystallographic refinement, in which the empirical restraints are replaced by more accurate QM calculations for the site of interest (in this case, the full FeV cluster with its ligands and the surrounding Gln-176, His-180 and Phe-362 residues). Separate refinements have been done with different interpretations of the light monoatomic ligand, replacing S2B, N^3−^, NH^2−^, ${\text{NH}}_{2}^{ - }$, OH^−^ or O^2−^. The QM calculations provide information about the ideal structure of the cluster with the various ligands, an information that is lacking in standard crystallography. Thereby, a structure is obtained that is an optimum compromise between the crystallographic raw data and the QM calculations. Since the crystallographic information is fully employed, we can use standard crystallographic quality estimates (RSZD score and *mF*_o_ − *DF*_c_ difference maps) and QM measures (energies and geometries) to judge which ligand fits the crystallographic raw data best.

With this method, we show first that OH^−^ ligand fits the crystallographic data best. However, with a single conformation, there are still large volumes of unresolved densities around the FeV cluster, indicating significant amounts of remaining S2B at the cluster and of the non-flipped conformation of Gln-176 (estimated to 11–17% by standard occupancy refinement with Phenix). Therefore, we repeated the quantum refinement with our new approach to allow for dual conformations in the QM system [50]. This gave significantly improved difference-density maps, but still showed that the OH^−^ ligand fits the crystallographic raw data significantly better than any N_2_-derived ligand.

Thus, our calculations indicate that the crystal structure shows an OH^−^-bound complex [66], rather than an N_2_-derived reaction intermediate. This agrees with the suggestion reached by Bjornsson and coworkers [28]. However, our calculations are appreciably more accurate, employing the crystallographic raw data both when obtaining the structures and when judging the various ligands. In particular, comparing Fe–Fe and Fe–S distances between a crystal structure and QM/MM calculations becomes questionable when the crystal structure involves dual conformations and therefore actually is a mixture of two different states, as was illustrated by the results in Table 3.

Yet, the fact that the crystal structure shows an OH^−^-bound complex does not mean that the structure is uninteresting. It shows that S2B may reversibly dissociate from the active-site cluster and that a flip of Gln-176 can bring it at a position where it can form hydrogen bonds to the reaction intermediates. The implications of these findings remain to be explored.

## Electronic supplementary material

Below is the link to the electronic supplementary material.

Supplementary file1 (PDF 149 kb)
