# Supplementary material for: Does the crystal structure of vanadium nitrogenase contain a reaction intermediate? Evidence from quantum refinement
Source: J Biol Inorg Chem. 2020 Aug 27;25(6):847–61. doi: 10.1007/s00775-020-01813-z (PMC7511287; doi:10.1007/s00775-020-01813-z)
Supplement: Supplementary file 1 — Supplementary file1 (PDF 149 kb) [file 775_2020_1813_MOESM1_ESM.pdf]

Supporting Information

Does the crystal structure of vanadium nitrogenase contain a reaction intermediate? Evidence from quantum refinement

Lili Cao, Octav Caldararu and Ulf Ryde *

Department of Theoretical Chemistry, Lund University, Chemical Centre, P. O. Box 124, SE-221 00 Lund, Sweden

Correspondence to Ulf Ryde, E-mail: Ulf.Ryde@teokem.lu.se,

Tel: +46 – 46 2224502, Fax: +46 – 46 2228648

2020-05-12

Table S1. Results of the quantum-refinement calculations for V-nitrogenase also with other charge states than in Table 1 in the main article. The table shows RSZD scores for the ligand, Gln-176 and His-180 (Sum is the sum of these three values) for different interpretations of the ligand replacing S2B (*X*), protonation states of His-180 and spin states (*S*). The last column are the relative energies of the various spin states and HID and HIE conformations (always within the same ligand and charge state).

X	Charge	His-180	*S*	RSZD score				Δ*E*
				X	Gln	His	Sum	kJ/mol
N^{3-}	–4	HIE	2	21.9	9.1	2.0	33.0	0.7
			3	22.1	8.9	2.0	33.0	0.0
	–5	HIE	3/2	20.7	10.2	2.0	32.9	
	–8	HIE	0	20.2	12.8	2.3	35.3	10.6
			1	19.2	13.1	2.2	34.5	0.4
			2	19.6	13.0	2.2	34.8	0.0
NH^{2-}	–3	HID	2	18.5	8.0	2.0	28.5	76.4
			3	18.3	8.1	2.0	28.4	96.9
		HIE	2	18.6	8.4	2.0	29.0	0.0
			3	19.0	8.2	2.0	29.2	7.9
	–4	HID	3/2	17.7	9.2	2.0	28.9	94.0
		HIE	3/2	17.8	9.7	2.0	29.5	0.0
	–7	HID	0	17.7	12.3	1.9	31.9	94.0
			1	17.1	12.5	1.9	31.5	0.0
			2	17.1	12.3	2.1	31.5	157.4
		HIE	0	17.3	12.3	2.3	31.9	31.7
			1	17.0	13.0	2.2	32.2	0.0
			2	17.4	12.7	2.3	32.4	49.9
NH_2^-	–2	HID	2	12.4	9.4	1.9	23.7	0.0
			3	12.6	9.3	1.9	23.8	6.7
	–3	HID	3/2	12.2	9.9	2.0	24.1	
	–4	HID	2	12.4	10.8	2.1	25.3	
	–6	HID	0	11.8	12.6	1.9	26.3	37.5
			1	11.6	12.4	2.8	26.8	2.3
			2	11.8	12.4	1.9	26.1	0.0
OH^-	–2	HID	2	10.4	6.3	2.0	18.7	47.1
			3	10.8	6.2	2.0	19.0	57.6
		HIE	2	9.4	6.7	2.0	18.1	5.4
			3	9.2	7.3	2.0	18.5	0.0
	–3	HID	3/2	11.2	6.8	2.1	20.1	88.9
		HIE	3/2	8.0	7.7	2.0	17.7	0.0
	–4	HID	2	8.2	7.8	2.0	18.0	104.5
		HIE	2	8.3	9.2	2.1	19.6	0.0
	–5	HID	3/2	10.0	9.1	2.2	21.3	104.5
		HIE	3/2	9.7	10.3	2.2	22.2	0.0
	–6	HID	0	6.6	9.8	2.3	18.7	160.0
			1	7.8	9.7	2.2	19.7	110.3
			2	8.2	9.9	2.1	20.2	168.7
		HIE	0	7.6	10.3	2.2	20.1	49.0
			1	8.6	10.1	2.2	20.9	0.0
			2	7.5	10.2	2.2	19.9	5.1
O^{2-}	–3	HIE	2	16.9	8.1	1.9	26.9	0.0
			3	16.4	8.2	1.9	26.5	8.1
	–4	HIE	3/2	15.4	9.0	2.0	26.4	
	–6	HIE	3/2	16.2	11.3	2.1	29.6	
	–7	HID	0	15.6	11.1	2.1	28.8	174.4
			1	15.0	11.1	2.2	28.3	139.6
			2	13.3	11.1	2.2	26.6	186.5
		HIE	0	14.6	11.6	2.2	28.4	37.5
			1	13.6	11.5	2.1	27.2	0.0
			2	14.0	11.6	2.1	27.7	3.1

Table S2. Coordinates of the ComQumX-2QM refined structures (pdb files of the QM coordinates).

OH$^-$–HIE, AC1

```
REMARK Energies (QM/MM, QM+ptch, MM3) =  -14958.999666 -15122.066050    219.530011 H
REMARK /lunarc/nobackup/projects/snic2019-35-66/Ulf/V-cryst/Cqx2qm/Oh/Store/Occ14
REMARK Fri Apr 10 05:41:23 CEST 2020
ATOM      1  H   ???     1      115.126  24.099 168.043
ATOM      2  C   ???     1      115.154  24.219 166.936
ATOM      3  H   ???     1      116.050  23.668 166.586
ATOM      4  H   ???     1      114.268  23.708 166.509
ATOM      5  C   ???     1      115.235  25.673 166.437
ATOM      6  H   ???     1      114.285  26.212 166.611
ATOM      7  H   ???     1      116.032  26.229 166.961
ATOM      8  C   ???     1      115.601  25.655 164.938
ATOM      9  O   ???     1      116.793  25.531 164.593
ATOM     10  N   ???     1      114.578  25.648 164.072
ATOM     11  H   ???     1      114.641  25.546 163.038
ATOM     12  H   ???     1      113.616  25.769 164.385
ATOM     13  H   ???     1      121.347  25.945 168.439
ATOM     14  C   ???     1      120.550  26.106 167.704
ATOM     15  C   ???     1      120.035  25.339 166.669
ATOM     16  H   ???     1      120.254  24.337 166.293
ATOM     17  N   ???     1      119.921  27.333 167.754
ATOM     18  C   ???     1      119.033  27.310 166.765
ATOM     19  H   ???     1      118.372  28.126 166.458
ATOM     20  N   ???     1      119.063  26.129 166.085
ATOM     21  H   ???     1      118.334  25.852 165.390
ATOM     22  H   ???     1      124.142  33.299 163.278
ATOM     23  C   ???     1      123.713  32.343 162.913
ATOM     24  H   ???     1      123.508  32.419 161.827
ATOM     25  H   ???     1      124.432  31.513 163.092
ATOM     26  S   ???     1      122.135  32.169 163.878
ATOM     27  H   ???     1      113.679  29.360 168.453
ATOM     28  C   ???     1      114.303  29.442 167.557
ATOM     29  C   ???     1      115.567  30.054 167.590
ATOM     30  H   ???     1      115.967  30.449 168.537
ATOM     31  C   ???     1      113.802  28.935 166.347
ATOM     32  H   ???     1      112.809  28.461 166.322
ATOM     33  C   ???     1      116.317  30.167 166.406
ATOM     34  H   ???     1      117.301  30.653 166.390
ATOM     35  C   ???     1      114.549  29.053 165.164
ATOM     36  H   ???     1      114.174  28.690 164.198
ATOM     37  C   ???     1      115.805  29.676 165.195
ATOM     38  H   ???     1      116.387  29.790 164.275
ATOM     39  H   ???     1      113.165  29.445 156.407
ATOM     40  C   ???     1      112.637  29.705 157.332
ATOM     41  C   ???     1      111.404  30.311 157.432
ATOM     42  H   ???     1      110.682  30.631 156.679
ATOM     43  N   ???     1      113.111  29.412 158.600
ATOM     44  C   ???     1      112.180  29.813 159.452
ATOM     45  H   ???     1      112.252  29.713 160.537
ATOM     46  N   ???     1      111.129  30.387 158.790
ATOM     47  H   ???     1      110.210  30.545 159.198
ATOM     48 FE   ???     1      120.692  30.946 162.548
ATOM     49  S   ???     1      118.787  31.107 163.886
ATOM     50  S   ???     1      120.886  28.729 162.193
ATOM     51  S   ???     1      120.335  31.846 160.442
ATOM     52 FE   ???     1      118.703  29.158 162.766
ATOM     53 FE   ???     1      119.768  29.650 160.403
ATOM     54 FE   ???     1      118.340  31.500 161.623
ATOM     55  C   ???     1      117.825  29.691 161.013
ATOM     56  S   ???     1      119.732  28.539 158.480
ATOM     57 FE   ???     1      116.065  30.464 160.408
ATOM     58 FE   ???     1      116.556  28.219 161.574
ATOM     59 FE   ???     1      117.648  28.664 159.291
ATOM     60  S   ???     1      114.516  29.157 161.494
ATOM     61  S   ???     1      116.635  26.712 159.919
ATOM     62  S   ???     1      116.120  29.919 158.226
ATOM     63  V   ???     1      114.916  28.261 159.331
ATOM     64  O   ???     1      117.422  27.773 163.285
ATOM     65  H   ???     1      117.537  26.806 163.394
ATOM     66  O   ???     1      109.164  25.859 156.657
ATOM     67  C   ???     1      109.573  26.524 157.653
ATOM     68  O   ???     1      108.875  27.043 158.576
ATOM     69  C   ???     1      111.114  26.698 157.801
ATOM     70  H   ???     1      111.582  26.832 156.805
```

```
ATOM     71  H   ???     1      111.310  27.589 158.419
ATOM     72  C   ???     1      111.758  25.490 158.491
ATOM     73  H   ???     1      111.214  25.278 159.435
ATOM     74  H   ???     1      111.670  24.602 157.837
ATOM     75  C   ???     1      113.252  25.681 158.838
ATOM     76  O   ???     1      113.401  26.788 159.763
ATOM     77  H   ???     1      113.384  26.339 160.699
ATOM     78  C   ???     1      114.074  26.018 157.577
ATOM     79  O   ???     1      114.074  25.222 156.626
ATOM     80  O   ???     1      114.723  27.136 157.624
ATOM     81  C   ???     1      113.835  24.414 159.506
ATOM     82  H   ???     1      113.720  23.564 158.812
ATOM     83  H   ???     1      114.914  24.605 159.685
ATOM     84  C   ???     1      113.147  24.117 160.856
ATOM     85  O   ???     1      112.587  23.021 161.049
ATOM     86  O   ???     1      113.206  25.104 161.703
ATOM     87  C   ???     1      116.043  33.274 161.297
ATOM     88  O   ???     1      115.382  32.246 160.778
ATOM     89  O   ???     1      117.354  33.139 161.470
ATOM     90  O   ???     1      115.468  34.340 161.586
END

AC2
REMARK Energies (QM/MM, QM+ptch, MM3) =  -14958.999666 -15444.158570    219.530011 H
REMARK /lunarc/nobackup/projects/snic2019-35-66/Ulf/V-cryst/Cqx2qm/Oh/Store/Occ14
REMARK Fri Apr 10 05:41:23 CEST 2020
ATOM      1  H   ???     1      115.142  24.224 167.990
ATOM      2  C   ???     1      115.200  24.528 166.907
ATOM      3  H   ???     1      114.655  25.490 166.842
ATOM      4  H   ???     1      116.259  24.787 166.705
ATOM      5  C   ???     1      114.701  23.608 165.793
ATOM      6  H   ???     1      114.931  24.086 164.820
ATOM      7  H   ???     1      115.238  22.632 165.793
ATOM      8  C   ???     1      113.209  23.332 165.888
ATOM      9  O   ???     1      112.633  23.486 166.977
ATOM     10  N   ???     1      112.584  22.892 164.754
ATOM     11  H   ???     1      111.565  22.869 164.868
ATOM     12  H   ???     1      112.865  23.154 163.742
ATOM     13  H   ???     1      121.378  25.959 168.440
ATOM     14  C   ???     1      120.672  26.153 167.620
ATOM     15  C   ???     1      120.186  25.472 166.508
ATOM     16  H   ???     1      120.329  24.460 166.122
ATOM     17  N   ???     1      120.193  27.452 167.633
ATOM     18  C   ???     1      119.434  27.555 166.544
ATOM     19  H   ???     1      118.913  28.445 166.172
ATOM     20  N   ???     1      119.398  26.392 165.842
ATOM     21  H   ???     1      118.859  26.345 164.929
ATOM     22  H   ???     1      124.151  33.286 163.275
ATOM     23  C   ???     1      123.735  32.321 162.911
ATOM     24  H   ???     1      123.391  32.433 161.863
ATOM     25  H   ???     1      124.524  31.534 162.954
ATOM     26  S   ???     1      122.304  32.017 164.045
ATOM     27  H   ???     1      113.675  29.401 168.398
ATOM     28  C   ???     1      114.214  29.503 167.446
ATOM     29  C   ???     1      115.237  30.455 167.304
ATOM     30  H   ???     1      115.516  31.094 168.158
ATOM     31  C   ???     1      113.872  28.674 166.360
ATOM     32  H   ???     1      113.083  27.915 166.475
ATOM     33  C   ???     1      115.897  30.598 166.072
ATOM     34  H   ???     1      116.710  31.321 165.929
ATOM     35  C   ???     1      114.540  28.807 165.134
ATOM     36  H   ???     1      114.304  28.166 164.276
ATOM     37  C   ???     1      115.545  29.778 164.991
ATOM     38  H   ???     1      116.062  29.886 164.032
ATOM     39  H   ???     1      113.174  29.389 156.347
ATOM     40  C   ???     1      112.612  29.593 157.260
ATOM     41  C   ???     1      111.249  29.782 157.278
ATOM     42  H   ???     1      110.520  29.802 156.462
ATOM     43  N   ???     1      113.068  29.443 158.564
ATOM     44  C   ???     1      111.975  29.492 159.321
ATOM     45  H   ???     1      112.003  29.342 160.403
ATOM     46  N   ???     1      110.821  29.699 158.600
ATOM     47  H   ???     1      109.971  28.990 158.699
ATOM     48 FE   ???     1      120.605  30.938 162.799
ATOM     49  S   ???     1      118.740  31.080 164.179
ATOM     50  S   ???     1      120.853  28.728 162.415
ATOM     51  S   ???     1      120.281  31.937 160.722
ATOM     52 FE   ???     1      118.611  29.152 162.956
ATOM     53 FE   ???     1      119.672  29.727 160.661
```

```
ATOM     54 FE   ???     1      118.233  31.529 161.892
ATOM     55  C   ???     1      117.754  29.724 161.247
ATOM     56  S   ???     1      119.691  28.597 158.753
ATOM     57 FE   ???     1      116.067  30.555 160.578
ATOM     58 FE   ???     1      116.497  28.217 161.706
ATOM     59 FE   ???     1      117.578  28.724 159.516
ATOM     60  S   ???     1      114.460  29.249 161.566
ATOM     61  S   ???     1      116.573  26.722 159.989
ATOM     62  S   ???     1      116.121  29.987 158.341
ATOM     63  V   ???     1      114.931  28.328 159.455
ATOM     64  S   ???     1      117.396  27.366 163.633
ATOM     65  O   ???     1      108.903  25.720 157.403
ATOM     66  C   ???     1      109.568  26.677 157.874
ATOM     67  O   ???     1      109.049  27.778 158.315
ATOM     68  C   ???     1      111.106  26.535 157.922
ATOM     69  H   ???     1      111.452  26.176 156.934
ATOM     70  H   ???     1      111.584  27.506 158.103
ATOM     71  C   ???     1      111.646  25.557 158.983
ATOM     72  H   ???     1      111.130  25.714 159.952
ATOM     73  H   ???     1      111.450  24.514 158.662
ATOM     74  C   ???     1      113.168  25.744 159.256
ATOM     75  O   ???     1      113.355  26.942 160.032
ATOM     76  H   ???     1      113.418  26.628 161.020
ATOM     77  C   ???     1      113.937  25.958 157.914
ATOM     78  O   ???     1      113.900  25.060 157.050
ATOM     79  O   ???     1      114.525  27.098 157.800
ATOM     80  C   ???     1      113.738  24.539 160.055
ATOM     81  H   ???     1      113.510  23.601 159.518
ATOM     82  H   ???     1      114.839  24.675 160.095
ATOM     83  C   ???     1      113.257  24.477 161.525
ATOM     84  O   ???     1      112.882  23.356 162.004
ATOM     85  O   ???     1      113.321  25.562 162.176
ATOM     86  C   ???     1      116.009  33.367 161.399
ATOM     87  O   ???     1      115.425  32.360 160.763
ATOM     88  O   ???     1      117.235  33.168 161.865
ATOM     89  O   ???     1      115.437  34.469 161.539
END
```

OH^-–HIE, QM system enhanced with Lys-83, Arg-339 and Lys-361; AC1

```
REMARK Energies (QM/MM, QM+ptch, MM3) =  -15398.574940 -15560.425236    221.957066 H
REMARK /lunarc/nobackup/projects/snic2019-35-66/Ulf/V-cryst/Cqx2qm/Store/Oh-hie/Lys_arg
REMARK Tue Apr 28 15:11:43 CEST 2020
ATOM      1  H   ???     1      117.661  23.602 156.483
ATOM      2  C   ???     1      117.532  23.997 157.507
ATOM      3  H   ???     1      118.100  23.410 158.245
ATOM      4  H   ???     1      116.457  24.005 157.755
ATOM      5  N   ???     1      118.000  25.422 157.567
ATOM      6  H   ???     1      117.647  25.904 158.511
ATOM      7  H   ???     1      117.536  26.004 156.846
ATOM      8  H   ???     1      119.021  25.581 157.510
ATOM      9  H   ???     1      115.125  24.090 168.052
ATOM     10  C   ???     1      115.152  24.199 166.953
ATOM     11  H   ???     1      116.021  23.612 166.596
ATOM     12  H   ???     1      114.240  23.730 166.535
ATOM     13  C   ???     1      115.287  25.659 166.488
ATOM     14  H   ???     1      114.370  26.238 166.705
ATOM     15  H   ???     1      116.127  26.160 166.998
ATOM     16  C   ???     1      115.591  25.667 164.990
ATOM     17  O   ???     1      116.764  25.628 164.568
ATOM     18  N   ???     1      114.527  25.619 164.166
ATOM     19  H   ???     1      114.566  25.483 163.140
ATOM     20  H   ???     1      113.589  25.553 164.559
ATOM     21  H   ???     1      121.347  25.951 168.439
ATOM     22  C   ???     1      120.557  26.117 167.703
ATOM     23  C   ???     1      120.059  25.346 166.668
ATOM     24  H   ???     1      120.292  24.346 166.298
ATOM     25  N   ???     1      119.913  27.337 167.754
ATOM     26  C   ???     1      119.026  27.305 166.771
ATOM     27  H   ???     1      118.342  28.109 166.483
ATOM     28  N   ???     1      119.077  26.125 166.083
ATOM     29  H   ???     1      118.370  25.831 165.389
ATOM     30  H   ???     1      124.143  33.299 163.280
ATOM     31  C   ???     1      123.716  32.342 162.916
ATOM     32  H   ???     1      123.523  32.411 161.830
ATOM     33  H   ???     1      124.415  31.508 163.122
ATOM     34  S   ???     1      122.128  32.161 163.873
ATOM     35  H   ???     1      117.245  35.092 158.710
ATOM     36  C   ???     1      116.812  34.256 158.135
ATOM     37  H   ???     1      116.596  34.580 157.102
```

ATOM	38	H	???	1	115.899	33.891	158.642
ATOM	39	N	???	1	117.842	33.210	158.110
ATOM	40	H	???	1	118.304	33.011	159.017
ATOM	41	C	???	1	118.017	32.302	157.152
ATOM	42	N	???	1	117.193	32.309	156.048
ATOM	43	H	???	1	116.197	32.375	156.288
ATOM	44	H	???	1	117.365	31.517	155.424
ATOM	45	N	???	1	119.077	31.468	157.187
ATOM	46	H	???	1	119.653	31.540	158.049
ATOM	47	H	???	1	118.913	30.459	157.004
ATOM	48	H	???	1	110.412	28.600	163.677
ATOM	49	C	???	1	111.492	28.455	163.409
ATOM	50	H	???	1	112.026	28.016	164.264
ATOM	51	H	???	1	111.957	29.427	163.173
ATOM	52	N	???	1	111.735	27.551	162.211
ATOM	53	H	???	1	111.998	26.525	162.359
ATOM	54	H	???	1	112.600	27.934	161.718
ATOM	55	H	???	1	110.975	27.561	161.498
ATOM	56	H	???	1	113.682	29.362	168.452
ATOM	57	C	???	1	114.308	29.445	167.562
ATOM	58	C	???	1	115.564	30.067	167.606
ATOM	59	H	???	1	115.950	30.464	168.555
ATOM	60	C	???	1	113.820	28.931	166.351
ATOM	61	H	???	1	112.834	28.444	166.333
ATOM	62	C	???	1	116.323	30.189	166.432
ATOM	63	H	???	1	117.302	30.685	166.440
ATOM	64	C	???	1	114.581	29.054	165.180
ATOM	65	H	???	1	114.238	28.665	164.215
ATOM	66	C	???	1	115.827	29.692	165.219
ATOM	67	H	???	1	116.415	29.802	164.304
ATOM	68	H	???	1	113.164	29.439	156.408
ATOM	69	C	???	1	112.640	29.691	157.334
ATOM	70	C	???	1	111.408	30.301	157.420
ATOM	71	H	???	1	110.694	30.611	156.657
ATOM	72	N	???	1	113.114	29.417	158.607
ATOM	73	C	???	1	112.181	29.853	159.447
ATOM	74	H	???	1	112.280	29.867	160.531
ATOM	75	N	???	1	111.133	30.401	158.769
ATOM	76	H	???	1	110.248	30.693	159.179
ATOM	77	FE	???	1	120.694	30.951	162.556
ATOM	78	S	???	1	118.790	31.103	163.881
ATOM	79	S	???	1	120.881	28.738	162.192
ATOM	80	S	???	1	120.322	31.855	160.435
ATOM	81	FE	???	1	118.700	29.157	162.775
ATOM	82	FE	???	1	119.768	29.656	160.413
ATOM	83	FE	???	1	118.337	31.502	161.630
ATOM	84	C	???	1	117.810	29.695	161.006
ATOM	85	S	???	1	119.734	28.537	158.471
ATOM	86	FE	???	1	116.057	30.463	160.418
ATOM	87	FE	???	1	116.557	28.212	161.571
ATOM	88	FE	???	1	117.651	28.674	159.291
ATOM	89	S	???	1	114.501	29.137	161.491
ATOM	90	S	???	1	116.641	26.704	159.911
ATOM	91	S	???	1	116.119	29.928	158.229
ATOM	92	V	???	1	114.919	28.257	159.324
ATOM	93	O	???	1	117.413	27.796	163.246
ATOM	94	H	???	1	117.370	26.874	163.603
ATOM	95	O	???	1	109.160	25.897	156.712
ATOM	96	C	???	1	109.609	26.541	157.695
ATOM	97	O	???	1	108.972	27.045	158.669
ATOM	98	C	???	1	111.154	26.691	157.772
ATOM	99	H	???	1	111.592	26.769	156.757
ATOM	100	H	???	1	111.393	27.604	158.342
ATOM	101	C	???	1	111.771	25.494	158.494
ATOM	102	H	???	1	111.221	25.312	159.442
ATOM	103	H	???	1	111.671	24.587	157.869
ATOM	104	C	???	1	113.258	25.679	158.835
ATOM	105	O	???	1	113.418	26.793	159.770
ATOM	106	H	???	1	113.491	26.299	160.686
ATOM	107	C	???	1	114.083	26.013	157.583
ATOM	108	O	???	1	114.107	25.211	156.643
ATOM	109	O	???	1	114.744	27.132	157.628
ATOM	110	C	???	1	113.844	24.426	159.525
ATOM	111	H	???	1	113.738	23.561	158.851
ATOM	112	H	???	1	114.918	24.618	159.728
ATOM	113	C	???	1	113.125	24.139	160.859
ATOM	114	O	???	1	112.579	23.060	161.065
ATOM	115	O	???	1	113.162	25.172	161.701
ATOM	116	C	???	1	116.045	33.275	161.289

```
ATOM    117  O   ???     1      115.386  32.234 160.773
ATOM    118  O   ???     1      117.365  33.139 161.437
ATOM    119  O   ???     1      115.473  34.331 161.570
END

AC2
REMARK Energies (QM/MM, QM+ptch, MM3) =  -15398.574940 -15882.783545    221.957066 H
REMARK /lunarc/nobackup/projects/snic2019-35-66/Ulf/V-cryst/Cqx2qm/Store/Oh-hie/Lys_arg
REMARK Tue Apr 28 15:11:43 CEST 2020
ATOM      1  H   ???     1      117.234  23.604 156.434
ATOM      2  C   ???     1      117.185  23.966 157.475
ATOM      3  H   ???     1      118.129  23.768 158.011
ATOM      4  H   ???     1      116.334  23.497 157.994
ATOM      5  N   ???     1      116.925  25.422 157.437
ATOM      6  H   ???     1      116.842  25.867 158.453
ATOM      7  H   ???     1      115.964  25.574 157.007
ATOM      8  H   ???     1      117.653  25.977 156.960
ATOM      9  H   ???     1      115.145  24.198 168.005
ATOM     10  C   ???     1      115.206  24.465 166.923
ATOM     11  H   ???     1      114.740  25.464 166.828
ATOM     12  H   ???     1      116.281  24.610 166.694
ATOM     13  C   ???     1      114.598  23.518 165.898
ATOM     14  H   ???     1      114.872  23.861 164.879
ATOM     15  H   ???     1      115.009  22.489 165.992
ATOM     16  C   ???     1      113.085  23.437 166.003
ATOM     17  O   ???     1      112.494  23.843 167.005
ATOM     18  N   ???     1      112.441  22.860 164.928
ATOM     19  H   ???     1      111.422  22.954 164.986
ATOM     20  H   ???     1      112.790  23.007 163.957
ATOM     21  H   ???     1      121.367  25.953 168.426
ATOM     22  C   ???     1      120.662  26.133 167.609
ATOM     23  C   ???     1      120.213  25.398 166.522
ATOM     24  H   ???     1      120.403  24.382 166.171
ATOM     25  N   ???     1      120.130  27.409 167.585
ATOM     26  C   ???     1      119.370  27.453 166.500
ATOM     27  H   ???     1      118.803  28.308 166.123
ATOM     28  N   ???     1      119.386  26.264 165.829
ATOM     29  H   ???     1      118.892  26.135 164.930
ATOM     30  H   ???     1      124.151  33.286 163.274
ATOM     31  C   ???     1      123.739  32.318 162.907
ATOM     32  H   ???     1      123.459  32.415 161.841
ATOM     33  H   ???     1      124.495  31.515 163.021
ATOM     34  S   ???     1      122.248  32.028 163.969
ATOM     35  H   ???     1      117.239  35.083 158.755
ATOM     36  C   ???     1      116.782  34.171 158.340
ATOM     37  H   ???     1      116.557  34.313 157.268
ATOM     38  H   ???     1      115.876  33.893 158.907
ATOM     39  N   ???     1      117.818  33.143 158.473
ATOM     40  H   ???     1      118.235  33.024 159.417
ATOM     41  C   ???     1      118.134  32.223 157.563
ATOM     42  N   ???     1      117.345  32.099 156.434
ATOM     43  H   ???     1      116.356  31.956 156.692
ATOM     44  H   ???     1      117.654  31.308 155.861
ATOM     45  N   ???     1      119.268  31.508 157.661
ATOM     46  H   ???     1      119.814  31.704 158.528
ATOM     47  H   ???     1      119.206  30.459 157.568
ATOM     48  H   ???     1      110.553  28.809 163.637
ATOM     49  C   ???     1      111.615  28.894 163.372
ATOM     50  H   ???     1      112.255  28.750 164.256
ATOM     51  H   ???     1      111.824  29.894 162.955
ATOM     52  N   ???     1      111.996  27.894 162.346
ATOM     53  H   ???     1      112.928  28.254 161.935
ATOM     54  H   ???     1      111.352  27.858 161.539
ATOM     55  H   ???     1      112.223  26.898 162.642
ATOM     56  H   ???     1      113.684  29.407 168.392
ATOM     57  C   ???     1      114.219  29.513 167.440
ATOM     58  C   ???     1      115.127  30.564 167.247
ATOM     59  H   ???     1      115.310  31.284 168.058
ATOM     60  C   ???     1      114.008  28.572 166.416
ATOM     61  H   ???     1      113.328  27.723 166.581
ATOM     62  C   ???     1      115.792  30.707 166.020
ATOM     63  H   ???     1      116.507  31.522 165.852
ATOM     64  C   ???     1      114.692  28.704 165.198
ATOM     65  H   ???     1      114.585  27.962 164.397
ATOM     66  C   ???     1      115.565  29.783 164.995
ATOM     67  H   ???     1      116.096  29.880 164.044
ATOM     68  H   ???     1      113.174  29.417 156.397
ATOM     69  C   ???     1      112.640  29.648 157.326
ATOM     70  C   ???     1      111.312  30.046 157.390
```

```
ATOM     71  H   ???     1      110.611  30.229 156.567
ATOM     72  N   ???     1      113.109  29.492 158.621
ATOM     73  C   ???     1      112.032  29.798 159.389
ATOM     74  H   ???     1      112.111  29.846 160.477
ATOM     75  N   ???     1      110.906  30.118 158.709
ATOM     76  H   ???     1      109.755  28.742 158.464
ATOM     77 FE   ???     1      120.628  30.920 162.776
ATOM     78  S   ???     1      118.726  31.076 164.086
ATOM     79  S   ???     1      120.846  28.718 162.358
ATOM     80  S   ???     1      120.284  31.896 160.675
ATOM     81 FE   ???     1      118.641  29.157 162.913
ATOM     82 FE   ???     1      119.673  29.680 160.624
ATOM     83 FE   ???     1      118.249  31.520 161.846
ATOM     84  C   ???     1      117.757  29.711 161.204
ATOM     85  S   ???     1      119.612  28.516 158.671
ATOM     86 FE   ???     1      116.121  30.569 160.511
ATOM     87 FE   ???     1      116.510  28.264 161.760
ATOM     88 FE   ???     1      117.548  28.678 159.524
ATOM     89  S   ???     1      114.514  29.346 161.589
ATOM     90  S   ???     1      116.478  26.698 160.109
ATOM     91  S   ???     1      116.119  29.921 158.303
ATOM     92  V   ???     1      114.863  28.388 159.460
ATOM     93  S   ???     1      117.431  27.397 163.583
ATOM     94  O   ???     1      109.066  25.870 157.519
ATOM     95  C   ???     1      109.715  26.832 157.904
ATOM     96  O   ???     1      109.107  28.004 158.176
ATOM     97  C   ???     1      111.220  26.803 158.102
ATOM     98  H   ???     1      111.688  26.850 157.100
ATOM     99  H   ???     1      111.558  27.700 158.645
ATOM    100  C   ???     1      111.727  25.564 158.847
ATOM    101  H   ???     1      111.145  25.407 159.778
ATOM    102  H   ???     1      111.615  24.656 158.227
ATOM    103  C   ???     1      113.211  25.736 159.237
ATOM    104  O   ???     1      113.355  26.932 160.033
ATOM    105  H   ???     1      113.524  26.553 160.995
ATOM    106  C   ???     1      114.067  25.950 157.972
ATOM    107  O   ???     1      114.263  24.990 157.180
ATOM    108  O   ???     1      114.580  27.111 157.812
ATOM    109  C   ???     1      113.724  24.527 160.066
ATOM    110  H   ???     1      113.479  23.586 159.546
ATOM    111  H   ???     1      114.826  24.626 160.146
ATOM    112  C   ???     1      113.188  24.486 161.518
ATOM    113  O   ???     1      112.787  23.416 162.006
ATOM    114  O   ???     1      113.256  25.622 162.144
ATOM    115  C   ???     1      116.068  33.362 161.307
ATOM    116  O   ???     1      115.495  32.356 160.643
ATOM    117  O   ???     1      117.350  33.195 161.638
ATOM    118  O   ???     1      115.469  34.417 161.530
END
```

OH⁻–HID, AC1

```
REMARK Energies (QM/MM, QM+ptch, MM3) =  -14958.965570 -15122.026351    219.530011 H
REMARK /lunarc/nobackup/projects/snic2019-35-66/Ulf/V-cryst/Cqx2qm/Oh/Store/Hid
REMARK Thu Apr  9 20:31:03 CEST 2020
ATOM      1  H   ???     1      115.125  24.097 168.046
ATOM      2  C   ???     1      115.153  24.214 166.940
ATOM      3  H   ???     1      116.084  23.723 166.589
ATOM      4  H   ???     1      114.299  23.653 166.510
ATOM      5  C   ???     1      115.154  25.669 166.448
ATOM      6  H   ???     1      114.179  26.156 166.647
ATOM      7  H   ???     1      115.937  26.257 166.957
ATOM      8  C   ???     1      115.503  25.675 164.940
ATOM      9  O   ???     1      116.685  25.588 164.598
ATOM     10  N   ???     1      114.459  25.666 164.089
ATOM     11  H   ???     1      114.514  25.556 163.056
ATOM     12  H   ???     1      113.500  25.751 164.419
ATOM     13  H   ???     1      121.356  25.948 168.455
ATOM     14  C   ???     1      120.587  26.112 167.712
ATOM     15  C   ???     1      120.098  25.358 166.665
ATOM     16  H   ???     1      120.387  24.346 166.363
ATOM     17  N   ???     1      119.882  27.308 167.653
ATOM     18  C   ???     1      119.023  27.222 166.577
ATOM     19  H   ???     1      118.387  28.047 166.239
ATOM     20  N   ???     1      119.136  26.053 165.983
ATOM     21  H   ???     1      120.068  28.149 168.199
ATOM     22  H   ???     1      124.143  33.298 163.279
ATOM     23  C   ???     1      123.714  32.343 162.914
ATOM     24  H   ???     1      123.505  32.418 161.829
ATOM     25  H   ???     1      124.435  31.514 163.090
```

```
ATOM     26  S   ???     1      122.136  32.169 163.881
ATOM     27  H   ???     1      113.681  29.361 168.454
ATOM     28  C   ???     1      114.306  29.444 167.559
ATOM     29  C   ???     1      115.568  30.063 167.593
ATOM     30  H   ???     1      115.959  30.467 168.540
ATOM     31  C   ???     1      113.811  28.933 166.349
ATOM     32  H   ???     1      112.822  28.451 166.324
ATOM     33  C   ???     1      116.319  30.175 166.409
ATOM     34  H   ???     1      117.299  30.672 166.389
ATOM     35  C   ???     1      114.560  29.049 165.168
ATOM     36  H   ???     1      114.191  28.673 164.204
ATOM     37  C   ???     1      115.812  29.677 165.198
ATOM     38  H   ???     1      116.399  29.785 164.280
ATOM     39  H   ???     1      113.165  29.446 156.406
ATOM     40  C   ???     1      112.637  29.706 157.332
ATOM     41  C   ???     1      111.403  30.311 157.432
ATOM     42  H   ???     1      110.681  30.631 156.680
ATOM     43  N   ???     1      113.110  29.413 158.599
ATOM     44  C   ???     1      112.180  29.813 159.452
ATOM     45  H   ???     1      112.253  29.712 160.536
ATOM     46  N   ???     1      111.128  30.387 158.790
ATOM     47  H   ???     1      110.208  30.540 159.198
ATOM     48 FE   ???     1      120.692  30.945 162.548
ATOM     49  S   ???     1      118.788  31.108 163.890
ATOM     50  S   ???     1      120.888  28.731 162.193
ATOM     51  S   ???     1      120.336  31.847 160.442
ATOM     52 FE   ???     1      118.702  29.156 162.767
ATOM     53 FE   ???     1      119.768  29.650 160.403
ATOM     54 FE   ???     1      118.340  31.500 161.624
ATOM     55  C   ???     1      117.827  29.694 161.013
ATOM     56  S   ???     1      119.733  28.540 158.480
ATOM     57 FE   ???     1      116.065  30.464 160.408
ATOM     58 FE   ???     1      116.558  28.216 161.576
ATOM     59 FE   ???     1      117.648  28.664 159.291
ATOM     60  S   ???     1      114.517  29.157 161.494
ATOM     61  S   ???     1      116.635  26.714 159.916
ATOM     62  S   ???     1      116.120  29.919 158.226
ATOM     63  V   ???     1      114.916  28.260 159.331
ATOM     64  O   ???     1      117.425  27.790 163.275
ATOM     65  H   ???     1      117.473  26.862 163.610
ATOM     66  O   ???     1      109.162  25.860 156.656
ATOM     67  C   ???     1      109.573  26.525 157.652
ATOM     68  O   ???     1      108.874  27.043 158.574
ATOM     69  C   ???     1      111.113  26.698 157.800
ATOM     70  H   ???     1      111.581  26.833 156.804
ATOM     71  H   ???     1      111.310  27.589 158.418
ATOM     72  C   ???     1      111.758  25.491 158.490
ATOM     73  H   ???     1      111.215  25.279 159.434
ATOM     74  H   ???     1      111.670  24.602 157.835
ATOM     75  C   ???     1      113.252  25.681 158.836
ATOM     76  O   ???     1      113.401  26.787 159.762
ATOM     77  H   ???     1      113.377  26.334 160.698
ATOM     78  C   ???     1      114.073  26.019 157.576
ATOM     79  O   ???     1      114.073  25.222 156.624
ATOM     80  O   ???     1      114.723  27.136 157.622
ATOM     81  C   ???     1      113.834  24.413 159.504
ATOM     82  H   ???     1      113.723  23.564 158.807
ATOM     83  H   ???     1      114.912  24.603 159.687
ATOM     84  C   ???     1      113.140  24.115 160.850
ATOM     85  O   ???     1      112.586  23.016 161.047
ATOM     86  O   ???     1      113.186  25.107 161.690
ATOM     87  C   ???     1      116.043  33.274 161.298
ATOM     88  O   ???     1      115.382  32.247 160.779
ATOM     89  O   ???     1      117.354  33.140 161.471
ATOM     90  O   ???     1      115.468  34.342 161.587
END
```

NH^{2-}—HID, AC1

```
REMARK Energies (QM/MM, QM+ptch, MM3) =  -14941.592668 -15101.777510    221.581898 H
REMARK /lunarc/nobackup/projects/snic2019-35-66/Ulf/V-cryst/Cqx2qm/Oh/Store/Nh-hid
REMARK Sat Apr 11 21:35:22 CEST 2020
ATOM      1  H   ???     1      115.124  24.094 168.050
ATOM      2  C   ???     1      115.151  24.207 166.948
ATOM      3  H   ???     1      116.081  23.720 166.594
ATOM      4  H   ???     1      114.295  23.649 166.518
ATOM      5  C   ???     1      115.147  25.667 166.473
ATOM      6  H   ???     1      114.174  26.150 166.687
ATOM      7  H   ???     1      115.935  26.249 166.981
ATOM      8  C   ???     1      115.483  25.679 164.969
```

ATOM	9	O	???	1	116.661	25.615	164.613
ATOM	10	N	???	1	114.431	25.657	164.123
ATOM	11	H	???	1	114.486	25.533	163.094
ATOM	12	H	???	1	113.473	25.700	164.465
ATOM	13	H	???	1	121.358	25.948	168.455
ATOM	14	C	???	1	120.591	26.111	167.711
ATOM	15	C	???	1	120.105	25.374	166.652
ATOM	16	H	???	1	120.400	24.371	166.328
ATOM	17	N	???	1	119.882	27.305	167.677
ATOM	18	H	???	1	120.045	28.122	168.263
ATOM	19	C	???	1	119.015	27.231	166.609
ATOM	20	H	???	1	118.358	28.053	166.308
ATOM	21	N	???	1	119.133	26.076	165.987
ATOM	22	H	???	1	124.142	33.297	163.278
ATOM	23	C	???	1	123.714	32.340	162.914
ATOM	24	H	???	1	123.515	32.413	161.828
ATOM	25	H	???	1	124.425	31.509	163.103
ATOM	26	S	???	1	122.131	32.164	163.874
ATOM	27	H	???	1	113.678	29.360	168.456
ATOM	28	C	???	1	114.302	29.442	167.564
ATOM	29	C	???	1	115.562	30.062	167.600
ATOM	30	H	???	1	115.950	30.468	168.547
ATOM	31	C	???	1	113.807	28.930	166.355
ATOM	32	H	???	1	112.820	28.446	166.331
ATOM	33	C	???	1	116.314	30.174	166.419
ATOM	34	H	???	1	117.293	30.674	166.408
ATOM	35	C	???	1	114.560	29.040	165.178
ATOM	36	H	???	1	114.193	28.650	164.221
ATOM	37	C	???	1	115.810	29.672	165.209
ATOM	38	H	???	1	116.401	29.759	164.293
ATOM	39	H	???	1	113.167	29.445	156.408
ATOM	40	C	???	1	112.642	29.704	157.336
ATOM	41	C	???	1	111.413	30.317	157.435
ATOM	42	H	???	1	110.700	30.652	156.681
ATOM	43	N	???	1	113.112	29.407	158.605
ATOM	44	C	???	1	112.180	29.815	159.456
ATOM	45	H	???	1	112.238	29.712	160.540
ATOM	46	N	???	1	111.136	30.390	158.791
ATOM	47	H	???	1	110.234	30.611	159.205
ATOM	48	FE	???	1	120.693	30.945	162.549
ATOM	49	S	???	1	118.787	31.098	163.873
ATOM	50	S	???	1	120.874	28.727	162.191
ATOM	51	S	???	1	120.331	31.837	160.444
ATOM	52	FE	???	1	118.694	29.145	162.762
ATOM	53	FE	???	1	119.767	29.649	160.405
ATOM	54	FE	???	1	118.340	31.503	161.624
ATOM	55	C	???	1	117.852	29.698	160.929
ATOM	56	S	???	1	119.727	28.538	158.487
ATOM	57	FE	???	1	116.069	30.460	160.409
ATOM	58	FE	???	1	116.567	28.210	161.591
ATOM	59	FE	???	1	117.653	28.666	159.290
ATOM	60	S	???	1	114.528	29.151	161.488
ATOM	61	S	???	1	116.630	26.728	159.934
ATOM	62	S	???	1	116.118	29.916	158.230
ATOM	63	V	???	1	114.916	28.258	159.329
ATOM	64	N	???	1	117.468	27.815	163.110
ATOM	65	H	???	1	117.441	26.986	163.735
ATOM	66	O	???	1	109.175	25.869	156.665
ATOM	67	C	???	1	109.601	26.530	157.655
ATOM	68	O	???	1	108.892	27.042	158.573
ATOM	69	C	???	1	111.131	26.702	157.802
ATOM	70	H	???	1	111.597	26.837	156.805
ATOM	71	H	???	1	111.326	27.594	158.420
ATOM	72	C	???	1	111.769	25.492	158.494
ATOM	73	H	???	1	111.226	25.282	159.438
ATOM	74	H	???	1	111.682	24.603	157.841
ATOM	75	C	???	1	113.262	25.684	158.839
ATOM	76	O	???	1	113.415	26.788	159.769
ATOM	77	H	???	1	113.376	26.307	160.715
ATOM	78	C	???	1	114.078	26.022	157.582
ATOM	79	O	???	1	114.077	25.241	156.625
ATOM	80	O	???	1	114.741	27.142	157.640
ATOM	81	C	???	1	113.842	24.412	159.499
ATOM	82	H	???	1	113.723	23.565	158.803
ATOM	83	H	???	1	114.921	24.587	159.684
ATOM	84	C	???	1	113.144	24.120	160.846
ATOM	85	O	???	1	112.591	23.028	161.047
ATOM	86	O	???	1	113.189	25.127	161.675
ATOM	87	C	???	1	116.047	33.264	161.298

```
ATOM     88  O   ???     1      115.389  32.229 160.782
ATOM     89  O   ???     1      117.362  33.127 161.471
ATOM     90  O   ???     1      115.474  34.325 161.583
END
```
